# Investigation of novel circulating proteins, germ line single-nucleotide polymorphisms, and molecular tumor markers as potential efficacy biomarkers of first-line sunitinib therapy for advanced renal cell carcinoma

**DOI:** 10.1007/s00280-014-2539-0

**Published:** 2014-08-07

**Authors:** Robert J. Motzer, Thomas E. Hutson, Gary R. Hudes, Robert A. Figlin, Jean-Francois Martini, Patricia A. English, Xin Huang, Olga Valota, J. Andrew Williams

**Affiliations:** 1 Department of Medicine, Genitourinary Oncology Service, Memorial Sloan-Kettering Cancer Center, New York, NY USA; 2 GU Oncology Program, GU Center of Excellence, Baylor Sammons Cancer Center-Texas Oncology, Dallas, TX USA; 3 Department of Medical Oncology, Genitourinary Malignancies, Fox Chase Cancer Center, Philadelphia, PA USA; 4 Division of Hematology Oncology, Department of Medicine, Samuel Oschin Comprehensive Cancer Institute, Cedars-Sinai Medical Center, Los Angeles, CA USA; 5 Translational Oncology, Pfizer Oncology, La Jolla, CA USA; 6 Oncology Statistics, Pfizer Oncology, La Jolla, CA USA; 7 Clinical Development, Pfizer Oncology, Milan, Italy; 8 Translational Oncology, Pfizer Oncology, 10646 Science Center Drive, San Diego, CA 92121 USA

**Keywords:** Sunitinib, Renal cell carcinoma, Serum marker, Germ line polymorphism marker, Tumor marker

## Abstract

**Purpose:**

Sunitinib is a first-line advanced renal cell carcinoma (RCC) standard of care. In a randomized phase II trial comparing sunitinib treatment schedules, separate exploratory biomarker analyses investigated the correlations of efficacy with selected serum, germ line single-nucleotide polymorphism (SNP), or tumor markers.

**Methods:**

Advanced RCC patients received first-line sunitinib 50 mg/day on the approved 4-week-on-2-week-off schedule (*n* = 146) or 37.5 mg/day continuous dosing (*n* = 146). The following correlation analyses were performed: (1) response evaluation criteria in solid tumors-defined tumor response with serum soluble protein levels via two distinct multiplex (*n* < 1,000) platforms; (2) response and time-to-event outcomes with germ line SNPs in *vascular endothelial growth factor (VEGF)*-*A* and *VEGF receptor* (*VEGFR*)*3* genes; and (3) response and time-to-event outcomes with tumor immunohistochemistry status for hypoxia-inducible factor 1-alpha (HIF-1α) and carbonic anhydrase-IX or tumor *Von Hippel*–*Lindau* (*VHL*) gene inactivation status.

**Results:**

Lower baseline angiopoietin-2 (Ang-2) and higher baseline matrix metalloproteinase-2 (MMP-2) levels were identified by both platforms as statistically significantly associated with tumor response. There were no significant correlations between *VEGF*-*A* or *VEGFR3* SNPs and outcomes. Progression-free survival was longer for HIF-1α percent of tumor expression groups 0–2 (HIF-1α low) versus 3–4 (HIF-1α high; *p* = 0.034). There were no significant correlations between outcomes and each *VHL* inactivation mechanism [mutation (86 % of *VHL*-inactive patients), methylation (14 %), and large deletion (7 %)] or mechanisms combined.

**Conclusions:**

Serum Ang-2 and MMP-2 and tumor HIF-1α were identified as relevant baseline biomarkers of sunitinib activity in advanced RCC, warranting further research into their prognostic versus predictive value.

**Electronic supplementary material:**

The online version of this article (doi:10.1007/s00280-014-2539-0) contains supplementary material, which is available to authorized users.

## Introduction

Sunitinib malate (SUTENT^®^; Pfizer Inc., New York, NY, USA) is an orally administered small-molecule receptor tyrosine kinase (RTK) inhibitor of vascular endothelial growth factor receptors (VEGFRs), platelet-derived growth factor receptors (PDGFRs), stem cell growth factor receptor [kinase insert domain for tyrosine (KIT)], and other RTKs [1]. Therefore, sunitinib, approved worldwide for advanced renal cell carcinoma (RCC), is expected to reduce tumor growth and metastasis by inhibiting angiogenesis and cause regression by acting directly on cells expressing and dependent on these receptors.

A randomized, phase II study (Renal EFFECT Trial) was conducted in advanced RCC patients to characterize the difference in time to tumor progression (TTP; primary end point) of first-line sunitinib at the approved 50 mg/day on Schedule 4/2 (4 weeks on treatment, 2 weeks off) versus sunitinib 37.5 mg/day on continuous daily dosing (CDD) [2]. Median TTP was 9.9 versus 7.1 months, respectively (*p* = 0.090), with no statistically significant differences in overall survival (OS), tolerability, or patient-reported kidney cancer symptoms.

Although there are no validated biomarkers for targeted therapies in advanced RCC, emerging data on sunitinib, pazopanib, sorafenib, and bevacizumab present compelling hypotheses to test retrospectively in this study to better understand interpatient variability in clinical benefit. Previous studies have shown potential correlations between soluble protein baseline levels and efficacy for metastatic RCC (mRCC) patients receiving sunitinib [3, 4] and other VEGFR2 inhibitors, e.g., pazopanib [5] and sorafenib [6]. In addition, genetic variability, as germ line single-nucleotide polymorphisms (SNPs) in VEGF-related genes (e.g., *VEGF*-*A* and *VEGFR3*), has been investigated as a potential efficacy predictor with antiangiogenic agents [7–10]. This is based on the hypothesis that “host” (vs. tumor) characteristics influence endothelial cell function in tumor vasculature.

Finally, analyses of tumor samples from mRCC patients receiving sunitinib and other targeted agents via immunohistochemistry (IHC) and other methods have examined potential markers such as hypoxia-inducible factor 1-alpha (HIF-1α) [11–13].

Here, we report separate exploratory biomarker analyses conducted for the Renal EFFECT Trial to investigate potential correlations of efficacy with selected serum, germ line SNP, or tumor markers.

## Methods

### Study design

In this multicenter phase II study (*N* = 292), adult patients with histologically confirmed advanced RCC were randomized 1:1 to sunitinib 50 mg/day on Schedule 4/2 (*n* = 146) or 37.5 mg/day on CDD (*n* = 146) [2]. Randomization was stratified by risk factors based on published Memorial Sloan-Kettering Cancer Center (MSKCC) data [14]. Patients continued treatment up to 2 years or until disease progression, significant toxicity, or consent withdrawal.

The study was run in accordance with the International Conference on Harmonization Good Clinical Practice guidelines and applicable local regulatory requirements and laws, and approved by the institutional review board or independent ethics committee of each center (ClinicalTrials.gov: NCT00267748). All patients gave written informed consent.

### Analytical methods

#### Serum soluble proteins

Protein concentrations were quantified using two distinct multiplex platforms in a pilot study to correlate protein levels with best overall tumor response, per response evaluation criteria in solid tumors (RECIST) [15]: SOMAscan (SomaLogic, Inc., Boulder, CO, USA) and SearchLight (Aushon BioSystems, Inc., Billerica, MA, USA). The SOMAscan proteomic platform utilizes optimized aptamers and targets ~1,000 human proteins (Supplemental Table 1) [16]. The SearchLight platform is an antibody-based protein assay system that utilizes chemiluminescence detection to provide high sensitivity and accuracy [17]. Forty-five proteins were selected for assay using SearchLight based on their known or putative roles in angiogenesis and/or RCC biology (Supplemental Table 2), of which 39 were in the SOMAscan list. Analyzed samples were randomly selected from patients on Schedule 4/2 only.

#### Germ line SNPs

The TaqMan allelic discrimination procedure detected germ line SNPs from blood in *VEGF*-*A* and *VEGFR3* genes (Table 1) that were then analyzed to explore their potential associations with RECIST-defined tumor response, TTP, progression-free survival (PFS), and OS. Samples were analyzed from all patients with genotype data in both schedules combined (in this case, Caucasian patients only; 88 % of all patients).

**Table 1:** *VEGF*-*A* and *VEGFR3* SNPs assessed in this study

Gene	Description	Nucleotide position relative to start codon^a^	Region/amino acid change	Minor allele frequency, Caucasians, public data^b^ (%)	SNP ID	References
*VEGF*-*A*	−*2578 C/A*	−2056	Upstream of *VEGF*-*A*	41	*rs699947*	[8, 18, 19]
*VEGF*-*A*	−*1154 G/A*	−614	Promoter	33	*rs1570360*	[8, 19]
*VEGFR3*	*2670 C>G*	2670	*Exon 19, H890Q*	40	*rs448012*	[10]
*VEGFR3*	*3971 G>T*	3971	*Exon 30, R1324L*	9	*rs307821*	[10]
*VEGFR3*	*1480 A>G*	1480	*Exon 11, T484A*	8	*rs307826*	[10]

Prognostic markers provide information about a patient’s outcome, independent of therapy. Predictive markers provide information about response to a therapy. Adjustments for multiple testing were not always used in these studies

*SNP* Single-nucleotide polymorphism, *VEGF* vascular endothelial growth factor, *VEGFR* VEGF receptor

^a^NCBI transcript numbers used for calculating position relative to the start codon are *VEGF*-*A* NM_001025366, *VEGFR3* NM_182925

^b^Minor allele frequencies are from HapMap, dbSNP, or published data

#### Molecular tumor markers: IHC

Formalin-fixed paraffin-embedded (FFPE) blocks or unstained slides were requested from previously collected diagnostic nephrectomy or biopsy specimens at the time of original surgery for the molecular analysis of tumor specimens. These samples were used to analyze HIF-1α and carbonic anhydrase (CA)-IX expression and to explore their correlations with RECIST-defined tumor response, TTP, PFS, and OS. Mouse monoclonal HIF-1α antibody (catalog number NB100-123) and rabbit polyclonal CA-IX antibody (NB100-417; both Novus Biologicals; Littleton, CO, USA) were used to detect HIF-1α and CA-IX, respectively. Two separate attempts were made to find a reliable antibody for HIF-2α assessment. Even though a signal was detected on paraffin sections using Novus Biologicals mouse monoclonal antibody (clone EP190b; NB100-132) or rabbit polyclonal antibody (NB100-56632), the pathologist-reviewed staining was found to be nonspecific; therefore, HIF-2α testing was not conducted. IHC analyses were conducted at PhenoPath Laboratories (Seattle, WA, USA) under pathologist supervision. Tumor samples were analyzed from patients in both schedules separately and combined.

#### Molecular tumor markers: tumor *VHL* gene inactivation

The status of each tumor *VHL* gene inactivation mechanism, which included mutations, large deletions (copy number decreases), and methylation, was assessed to explore their correlation with RECIST-defined tumor response, TTP, PFS, and OS. Following pathologist review, deoxyribonucleic acid (DNA) from FFPE tissue was extracted using the QIAGEN FFPE DNA extraction kit protocol (QIAGEN Sciences Inc.; Germantown, MD, USA). Mutations in *VHL* gene exons 1–3 were detected using SURVEYOR^®^ and WAVE^®^ analyses, which can detect mutations in extracted DNA down to the level of 5 % heterozygosity (mutated VHL: normal VHL), and characterized by Sanger sequencing. Rules for predicting inactivation status were based on previously published criteria [20]. Laboratory analyses were conducted at Transgenomic, Inc. (Omaha, NE, USA). Intragenic large deletions in *VHL* and other surrounding genes on chromosome 3 were determined using multiplex ligation-dependent probe amplification (MLPA). The SALSA^®^ MLPA^®^ KIT P016-C2 *VHL* (MRC-Holland; Amsterdam, the Netherlands) was used to detect copy number variations. MLPA data analysis was conducted using GeneMarker^®^ version 1.85 (SoftGenetics; State College, PA, USA). *VHL* promoter region methylation status was assessed using bisulfite conversion of unmethylated cytosines followed by DNA sequencing with the EZ DNA Methylation-Gold™ Kit (Zymo Research Corporation; Irvine, CA, USA). To confirm methylation status, all samples were bidirectionally sequenced using second-round polymerase chain reaction primer sets and BIG DYE^®^, version 3.0 on an Applied Biosystems 3730XL instrument. For quality control, K562 (Promega; Madison, WI, USA), a human erythroleukemic cell line, and a universal methylated human DNA standard (Zymo Research Corporation; Irvine, CA, USA) were analyzed with the samples. Tumor samples were analyzed from patients in both schedules combined and Schedule 4/2 only.

### Statistical methods

#### Serum soluble proteins

The *p* values were determined by Wilcoxon rank-sum test and compared to unadjusted and Bonferroni-adjusted significance levels. *p* values were declared statistically significant if <0.05 (unadjusted comparison) or <0.000048 (adjusted comparison: 0.05/1,046 soluble proteins assayable). In addition, the false discovery rate (FDR) [21] for each *p* value was calculated. Accepting that a proportion of proteins at the 5 % significance level would be expected to be identified as false positives, the FDR was applied to provide an objective level of confidence to each assigned protein. RECIST-defined response was the primary outcome of interest in this pilot assessment.

#### Germ line SNPs

Potential correlations between five SNPs in *VEGF*-*A* (2 SNPs) and *VEGFR3* (3 SNPs) genes with TTP, PFS, and OS were evaluated by Kaplan–Meier method and a Cox proportional hazards model in which the independent variables were treatment and SNP genotype. A “genotype main effect” test was used to identify any relationships between TTP, PFS, or OS and genotype regardless of treatment, and an “interaction” test was used to identify any differences in the patterns of the genotype and TTP, PFS, or OS relationships between the two treatment arms. Fisher’s exact test was used to assess any potential correlation of genotype with objective response rate (ORR), and the log-rank test was used for time-to-event end points.

#### Molecular tumor markers: IHC

For IHC analysis of HIF-1α (nuclear staining observed) and CA-IX (cytoplasmic and membranous staining observed), molecular markers were scored by a single pathologist without image analysis and summarized as individual scores (percent of tumor expression—an estimation of the total tumor percent in the specimen; predominant intensity; focal score; and focal intensity) and composite scores (expression and focal composites; individual variable multiplied by expression intensity). Statistically significant *p* values (<0.05) were compared to unadjusted and Bonferroni-adjusted significance levels, where the adjustment was made based on eight individual molecular markers (HIF-1α percent of tumor expression; HIF-1α predominant intensity; HIF-1α focal score; HIF-1α focal intensity; CA-IX percent of tumor expression; CA-IX predominant intensity; CA-IX focal score; and CA-IX focal intensity). The four composite scores were highly correlated with the other markers so no adjustment was made for them. For the 12 comparisons, *p* values were declared significant if <0.05 (unadjusted) or <0.00625 (adjusted: 0.05/8 IHC tumor assay end points). TTP, PFS, and OS were compared between biomarker strata, based on a pre-specified cutoff point of three, in which the individual score was stratified in groups (0, 1, 2, 3, 4), for the HIF-1α and CA-IX expression level (i.e., 0: 0 % expression; 1: 1–25 % expression; 2: 26–50 % expression; 3: 51–75 % expression; and 4: 76–100 % expression) by Kaplan–Meier method, both within the individual treatment groups and both groups combined.

#### Molecular tumor markers: tumor VHL gene inactivation

The following *VHL* inactivation status markers were assessed. Overall *VHL* inactivation status could = no, yes, fail, or incomplete. The status = “yes” if at least one *VHL* status (mutational, methylation, or deletion) = “yes”; = “fail” if *VHL* mutational status was of unknown significance; = “no” if all three were “no”; and = “incomplete” if all tests were not successful. *p* values and significance levels for both unadjusted and adjusted comparisons were calculated. TTP, PFS, and OS were evaluated by Kaplan–Meier method and compared between strata defined by “yes”/“no” for each *VHL* gene inactivation variable in patients from both schedules combined. *p* values by unstratified log-rank test were compared to unadjusted and Bonferroni-adjusted significance levels.

For all analyses, all reported *p* values are unadjusted unless stated otherwise. The null hypothesis that markers are not associated with efficacy could be rejected if *p* values <0.05 were observed.

This study was powered to address efficacy and not biomarker end points; no post hoc power calculations were performed.

## Results

### Serum soluble proteins

All analyzed samples were collected from patients on Schedule 4/2. For this pilot study assessing two distinct multiplex platforms, 74 samples total were collected at baseline, and another 26 samples were collected at the end of treatment/withdrawal and had associated paired baseline values. Demographic characteristics (Supplemental Table 3) were similar between patients with complete/partial response (CR/PR) versus stable/progressive disease (SD/PD).

#### SearchLight platform

Only two proteins showed statistically significant (unadjusted *p* < 0.05) association with best overall response (CR/PR vs. SD/PD) at baseline: lower than median baseline angiopoietin-2 (Ang-2) serum concentrations (*p* = 0.0215) and higher than median baseline matrix metalloproteinase-2 (MMP-2) serum concentrations (*p* = 0.0180). However, at 0.9011, FDR for both was high (i.e., close to 1), indicating low confidence in the observed associations.

When responders (CR/PR) were compared to a combined group of patients with SD <24 weeks and/or PD (defined as tumor response extremes), three proteins showed potential association with increased response rate: lower baseline Ang-2 concentrations (*p* = 0.0236, FDR = 0.9816); lower baseline hepatocyte growth factor (HGF) concentrations (*p* = 0.0442, FDR = 0.9818); and higher baseline MMP-2 concentrations (*p* = 0.0233, FDR = 0.9818); however, for these three proteins, FDR was high (>0.98).

Six proteins were highly modulated by the end of treatment with an FDR ≤0.05. Levels of granulocyte colony-stimulating factor (G-CSF; unadjusted *p* = 0.0046, FDR = 0.0455); C-reactive protein (CRP; *p* = 0.0019, FDR = 0.0288); interleukin-6 (IL-6; *p* = 0.0046, FDR = 0.0455); MMP-1 (*p* = 0.0011, FDR = 0.0197); placenta growth factor (PlGF; unadjusted and adjusted *p* < 0.0001, FDR < 0.0001); and stem cell factor (SCF; *p* = 0.0006, FDR = 0.0146) increased by approximately 2.0-, 3.2-, 5.4-, 1.6-, 3.7-, and 1.7-fold relative to baseline levels, respectively.

Four proteins showed potential association between response and modulation at the end of treatment compared with baseline. Responders (CR/PR) had higher end of treatment relative to baseline levels of E-selectin (*p* = 0.0233); fibronectin (*p* = 0.0476); SCF (*p* = 0.0141); and sVEGFR2 (*p* = 0.0297); however, at 0.9657, the associated FDRs were high.

By stratifying more stringently by tumor response extremes, only SCF modulation at the end of treatment compared with baseline remained statistically significantly associated (*p* = 0.0089) with responders having a higher ratio of follow-up (end of treatment) to baseline levels than non-responders. However, FDR was 1.00, indicating very low confidence in the observed association.

#### SOMAscan platform

Using the broad SOMAscan analyte menu, 40 proteins showed potential association with best overall tumor response in serum samples collected at baseline (Table 2). Lower baseline serum concentrations associated with response were observed for ADAM metallopeptidase with thrombospondin type 1 motif, 4 (ADAMTS-4); aurora kinase B (AURKB); Ang-2; B-lymphocyte chemoattractant (BLC); CRP; CA-IV; Ck-b-8-1 [also referred to as myeloid progenitor inhibitory factor 1 (MPIF-1) splice variant 1]; Coactosin-like protein; Cripto; Cytochrome c; DEAD-box protein 19B (DDX19B); Granzyme B; Gro-g [also referred to as Chemokine (C–X–C motif) ligand 3 or CXCL3]; I-309 [also referred to as chemokine (C–C motif) ligand 1 or CCL1]; IL11 receptor alpha (IL-11 RA); MPIF-1; and Complement factor H-related 5. Higher baseline concentrations associated with response were observed for Afamin; Albumin; Angiogenin; Angiostatin; apolipoprotein E (Apo-E); Apo-E3; BGH3 (also referred to as transforming growth factor-beta-induced protein ig-h3); CD36 antigen; CD48; cell adhesion molecule-related/down-regulated by oncogenes (CDON); dickkopf 3 homolog (DKK3); Kallistatin; limbic system-associated membrane protein (LSAMP); MMP-2; mannose receptor C type 2 (MRC2); NCAM-120 (similar to neural cell adhesion molecule 1, 120-kDa isoform precursor); plexin C1 (PLXC1); secreted protein, acidic, rich in cysteine (SPARC or osteonectin)-like 1 (SPARCL1); thymus and activation-regulated chemokine (TARC, also known as CCL17); tissue inhibitor of metalloproteinase 2 (TIMP-2); tropomyosin-related kinase B (TrkB); WAP, kazal, immunoglobulin, kunitz, and NTR domain-containing protein 2 (WFKN2); and secreted frizzled-related protein 3 (sFRP-3). However, at 0.9011, FDR for all of these markers was high, indicating low confidence in the observed associations as noted previously.

**Table 2:** Soluble protein biomarkers from the SOMAscan platform with differences in baseline by best overall response in patients on Schedule 4/2, selected for unadjusted *p* value comparison ≤0.05

Analyte^a^	Units	Best overall tumor response (RECIST v1.0)						*p* value^b^
		CR or PR		*n*	SD or PD		*n*	
		Mean	Median		Mean	Median		
ADAMTS-4	pg/ml	247.41	238.00	27	264.91	249.00	44	0.0317
AURKB	pg/ml	639.04	625.00	27	663.55	662.50	44	0.0071
Afamin	μg/ml	104.58	109.00	27	83.87	91.20	44	0.0253
Albumin	μg/ml	1.04	1.07	27	0.92	0.93	44	0.0287
Angiogenin	ng/ml	457.33	464.00	27	406.75	408.00	44	0.0479
**Ang-2**	**ng/ml**	**29.63**	**29.60**	**27**	**40.08**	**35.00**	**44**	**0.0197**
Angiostatin	μg/ml	36.17	35.70	27	33.86	32.95	44	0.0318
Apo-E	μg/ml	57.27	57.10	27	48.40	49.40	44	0.0107
Apo-E3	μg/ml	31.82	30.90	27	27.01	27.20	44	0.0110
BGH3	μg/ml	2.60	2.31	27	2.13	2.15	44	0.0235
BLC	pg/ml	139.81	133.00	27	173.43	149.50	44	0.0228
CD36 antigen	ng/ml	70.49	71.30	27	65.50	61.00	44	0.0242
CD48	pg/ml	1,495.93	990.00	27	920.93	870.00	44	0.0218
CDON	ng/ml	38.50	35.60	27	32.78	31.30	44	0.0430
CRP	μg/ml	52.96	10.10	27	241.97	20.15	44	0.0485
CA-IV	ng/ml	17.27	17.20	27	17.76	17.90	44	0.0169
Ck-b-8-1	pg/ml	649.15	553.00	27	789.95	716.00	44	0.0235
Coactosin-like protein	ng/ml	2.55	2.52	27	2.75	2.74	44	0.0260
Cripto	ng/ml	2.46	2.30	27	2.81	2.71	44	0.0197
Cytochrome c	ng/ml	2.45	2.36	27	2.67	2.55	44	0.0360
DEAD-box protein 19B	ng/ml	149.63	149.00	27	175.82	158.00	44	0.0330
DKK3	ng/ml	28.57	28.60	27	26.25	25.55	44	0.0074
Granzyme B	pg/ml	99.84	99.80	27	126.25	104.50	44	0.0459
Gro-g	pg/ml	459.89	440.00	27	676.20	540.00	44	0.0442
I-309	pg/ml	118.70	118.00	27	123.38	123.00	44	0.0457
IL-11 RA	ng/ml	3.49	3.29	27	3.66	3.14	44	0.0067
Kallistatin	μg/ml	27.39	25.30	27	22.22	21.80	44	0.0442
LSAMP	ng/ml	9.44	9.40	27	8.64	7.94	44	0.0386
**MMP-2**	**ng/ml**	**9.21**	**9.07**	**27**	**8.25**	**7.87**	**44**	**0.0327**
MPIF-1	pg/ml	521.67	445.00	27	654.70	607.00	44	0.0253
MRC2	ng/ml	32.09	29.00	27	27.47	26.30	44	0.0268
NCAM-120	ng/ml	230.89	220.00	27	190.76	180.00	44	0.0016
PLXC1	ng/ml	2.46	2.22	27	1.93	1.88	44	0.0380
SPARCL1	ng/ml	369.04	367.00	27	331.32	321.00	44	0.0218
TARC	pg/ml	195.03	167.00	27	150.79	133.50	44	0.0164
TIMP-2	ng/ml	124.20	124.00	27	113.29	110.50	44	0.0155
TrkB	ng/ml	46.51	38.50	27	35.59	34.70	44	0.0396
WFKN2	ng/ml	12.94	13.10	27	11.93	11.55	44	0.0068
Complement factor H-related 5	μg/ml	2.71	2.68	27	3.26	3.11	44	0.0436
sFRP-3	ng/ml	10.62	7.81	27	6.85	6.58	44	0.0125

*CR* Complete response, *FDR* false discovery rate, *PD* progressive disease, *PR* partial response, *RECIST* response evaluation criteria in solid tumors, *SD* stable disease

^a^From a menu of ~980 analytes tested (see Supplemental Table 1). Analytes also identified by the SearchLight platform are in bold

^b^ Wilcoxon rank-sum test, with unadjusted *p* value comparison to *α* = 0.05; the adjusted comparison would be to *α* = 0.0000478; at 0.9011, the FDR for these markers was high (i.e., close to 1), indicating low confidence in the observed associations

When using the more stringent criteria of tumor response extremes, the SOMAscan analysis revealed 43 proteins that showed association with the response extreme in serum samples collected at baseline (summary statistics provided in Supplemental Table 4). Lower baseline concentrations of ADAMTS-4; AURKB; Ang-2; complement component 2 (C2); connective tissue-activating peptide III (CTAP-III, also known as CXCL7); Ck-b-8-1; Coactosin-like protein; Complement factor H-related 5; Cytochrome c; IL-11 RA; Kallikrein 5; lipopolysaccharide-binding protein (LBP); MPIF-1; plasminogen activator inhibitor type 1 (PAI-1); sialic acid-binding Ig-like lectin 9 (Siglec-9); and TIMP-1 were associated with extremes of response. Similarly, higher baseline concentrations of Afamin; Albumin; Apo-E; Apo-E3; Apo-E4; BGH3; CD36 antigen; CD48; Contactin-1; Contactin-5; DKK3; sFRP-3; Gelsolin; IL1 receptor accessory protein (IL-1R AcP); LSAMP; MMP-2; MRC2; NCAM-120; pancreatic polypeptide (or pro-hormone, PH); retinol-binding protein 4 (RBP); SPARCL1; TARC; TIMP-2; tryptase-beta 2 (TPSB2); TrkB; Trypsin; and WFKN2 were also associated with response extremes. However, for all of those proteins, FDR was high (>0.98, Supplemental Table 4).

Interestingly, on this platform, baseline HGF concentration did not appear to differentiate between response extremes (*p* = 0.6754).

Fifteen proteins were modulated during treatment (end-of-treatment samples compared with paired baseline samples) with unadjusted and adjusted *p* < 0.00009 and FDR < 0.005. For five of these proteins, serum levels increased following sunitinib treatment: CDK8/cyclin C; follistatin (FST); Factor I; MMP-2; and tissue factor pathway inhibitor (TFPI). In contrast, levels of IL-16; IL-31; insulin receptor (IR); immunoglobulin M (IgM); Karyopherin-a2; nephroblastoma overexpressed (NovH, also known as insulin-like growth factor binding protein 9); SCF sR; TPSB2; VEGF sR2; and sL-Selectin decreased following treatment. Another 86 analytes were also statistically significantly modulated, with unadjusted *p* ≤ 0.05 and FDR ≤ 0.05 (Supplemental Table 5).

Upon stratification by responders versus non-responders, 29 proteins showed potential association between modulation following treatment (end of treatment compared with baseline levels) and response (Supplemental Table 6). However, FDRs were high (0.9657), and none of the proteins identified overlapped between the two platforms. By stratifying more stringently by tumor response extremes, 36 proteins showed potential association between modulation following treatment and response; however, FDR was equal to 1.00 (Supplemental Table 7).

Hence, from this serum-based multiplex biomarker analysis, baseline Ang-2 and MMP-2 were the only proteins identified by both SearchLight and SOMAscan platforms as having a statistically significant association with best overall response.

### SNPs

Since only 70 % of patients donated blood samples for the germ line genetic analysis, it is important to understand the differences between the subpopulation with genotype data (*n* = 202; Supplemental Table 3) and the subpopulation that did not. There were no statistically significant differences between genotyped and non-genotyped patients in age, sex, race, or prognostic criteria (Karnofsky performance status [KPS] and risk group stratification based on MSKCC prognostic criteria). However, since marginal differences between genotyped and non-genotyped subpopulations in TTP (*p* = 0.0152), PFS (*p* = 0.062), and OS (*p* = 0.132) have been observed in favor of the genotyped subpopulation, this subpopulation may not be representative of the overall trial population. Therefore, caution is advised before extrapolating analyses for the genotyped patients to the full trial population.

Using the Cox proportional hazard model, there were no statistically significant associations below the 0.05 level for the “genotype main effect” test with TTP, PFS, or OS, with patients in both treatment arms combined. However, marginal statistically significant interactions between treatment and the *VEGFR3* SNP *rs448012* were observed for TTP, PFS, and OS (unadjusted *p* values and data not shown). A statistically significant interaction may indicate that the time-to-event (TTP, PFS, or OS) pattern among the genotypes is different between the two treatment arms. While any statistically significant interaction indicates differences in a time-to-event end point/genotype relationship between the two treatment arms, it is not designed to identify which, if any, treatment arm might have a statistically significant association between genotype and the time-to-event end point.

Table 3 lists hazard/odds ratios and *p* values (unadjusted) for the previously studied [10] *VEGFR3* SNP *rs307826* for each of the assessed efficacy end points to demonstrate those interactions.

**Table 3:** Hazard/odds ratios and unadjusted *p* values for the *VEGFR3* SNP *rs307826* for each of the efficacy end points assessed using Caucasian patients from both treatment arms combined

Efficacy end point	HR (95 % CI)	*p* value
TTP (*A*/*G* vs. *A*/*A*)	0.71 (0.41–1.24)	0.229
TTP (*G*/*G* vs. *A*/*A*)	1.00 (0.25–4.07)	1.00
PFS (*A*/*G* vs. *A*/*A*)	0.67 (0.39–1.15)	0.145
PFS (*G*/*G* vs. *A*/*A*)	0.94 (0.23–3.81)	0.929
OS (*G*/*C* vs. *G*/*G*)	1.01 (0.60–1.70)	0.974
OS (*C*/*C* vs. *G*/*G*)	1.30 (0.32–5.29)	0.714

Efficacy end point	OR (95 % CI)	*p* value
ORR (*G*/*C* vs. *A*/*A*)	1.00 (0.5–2.2)	1.00
ORR (*C*/*C* vs. *A*/*A*)	1.18 (0.1–13.4)	1.00

The homozygous wild-type *A/A* genotype was selected as reference genotype. An HR >1 indicates a reduction in risk in favor of the homozygous wild type. An OR >1 indicates a higher response rate in favor of the homozygous wild type

*CI* Confidence interval, *HR* hazard ratio, *OR* odds ratio, *ORR* objective response rate, *OS* overall survival, *PFS* progression-free survival, *SNP* single-nucleotide polymorphism, *TTP* time to tumor progression, *VEGFR* vascular endothelial growth factor receptor

### Molecular tumor markers: IHC

Age, weight, height, and race (Supplemental Table 3) were similar between the 149 IHC-evaluable patients (both schedules combined) and the overall trial population [2]; however, like the overall population, there was an imbalance in prognostic criteria between treatment arms: more patients in the CDD group had a low KPS of 70 % (12 vs. 3 %) and were classified as “poor risk” based on MSKCC prognostic criteria (14 vs. 8 %) than patients on Schedule 4/2 (16 patients total were classified as “poor risk” in both arms combined).

**Fig. 1:**
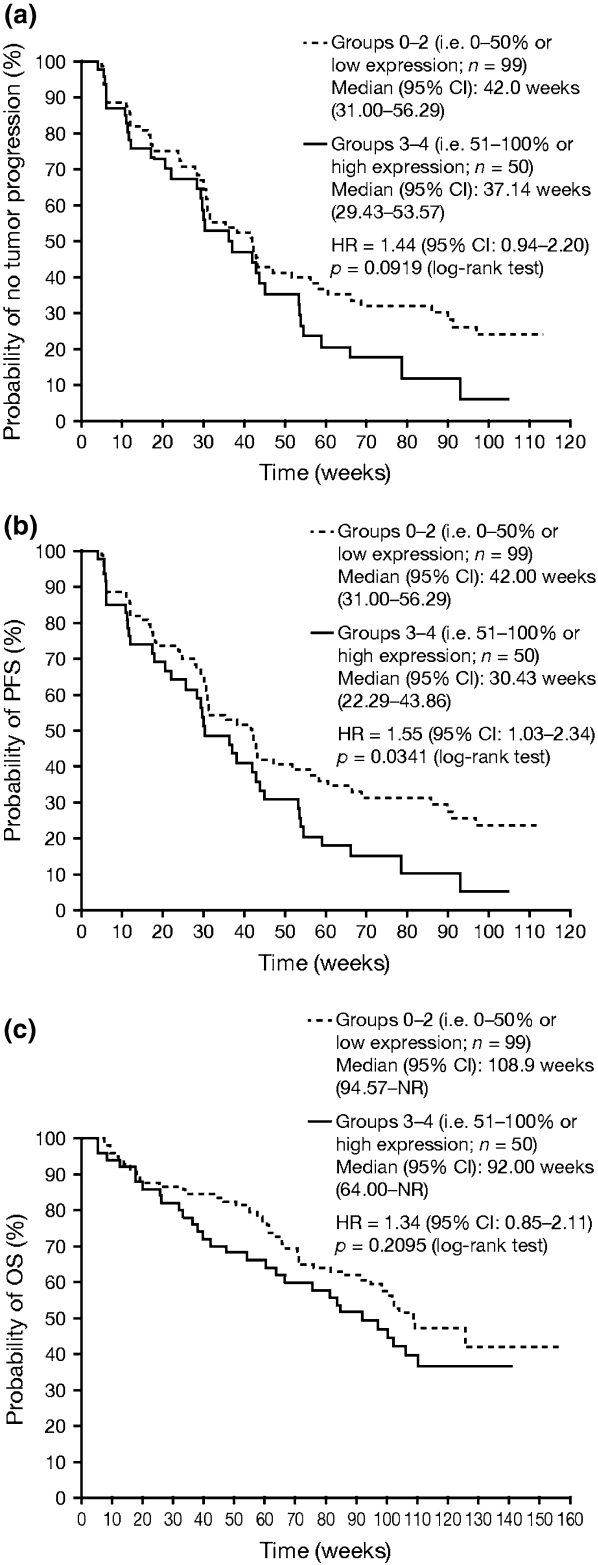
Kaplan–Meier curves of **a** TTP, **b** PFS, and **c** OS after stratification by level of HIF-1α percent of tumor expression in patients from both treatment arms combined (intent-to-treat population). HIF-1α percent of tumor expression (individual variable score) was stratified in groups (0, 1, 2, 3, 4) and the cutoff point was set at 3. Groups 0–2 correspond to 0–50 % (i.e., low HIF-1α expression) and groups 3–4 correspond to 51–100 % (i.e., high HIF-1α expression). An HR >1 indicates a reduction in hazard rate in favor of groups 0–2, whereas an HR <1 indicates a reduction in hazard rate in favor of groups 3–4. *HIF-1α* hypoxia-inducible factor 1-alpha, *HR* hazard ratio, *NR* not reached, *OS* overall survival, *PFS* progression-free survival, *TTP* time to tumor progression

For the combined treatment arm analysis, there were no statistically significant associations between TTP and OS with either HIF-1α or CA-IX percent of tumor expression, assessed by Kaplan–Meier analysis; similarly, no statistically significant association was observed between PFS and CA-IX percent of tumor expression. In contrast, in the analysis of HIF-1α percent of tumor expression, PFS was statistically significantly different between groups 0–2 (i.e., 0–50 % or low expression) versus groups 3–4 (i.e., 51–100 % or high expression) (*p* = 0.0341). Kaplan–Meier curves of TTP, PFS, and OS after stratification by groups 0–2 versus 3–4 for HIF-1α percent of tumor expression in patients from both treatment arms combined are shown in Fig. 1a–c, respectively. Although a trend can be observed in which lower HIF-1α percent of tumor expression appears to confer longer TTP (Fig. 1a) and OS (Fig. 1c), these associations were not statistically significant. Further analysis of each treatment arm separately indicated that lower HIF-1α percent of tumor expression was statistically significantly associated with improvements in both TTP and PFS in the Schedule 4/2 group; however, these associations were not statistically significant in the CDD group.

### Molecular tumor markers: tumor VHL gene inactivation

Of 143 patients evaluated for *VHL* inactivation status, 106 (74 %) had confirmed overall *VHL* inactivation status = “yes” (mutation and/or methylation and/or large deletion). Of those with overall *VHL* inactivation status = “yes,” 86 % had mutations, 7 % had large deletions, and 14 % had methylated CpG island status. Forty-three patients had mutations [single base changes or small insertions or small deletions or small insertions and deletions (indels)] that were not previously recorded in the COSMIC database and were therefore recorded as “Novel” mutations. Of the 143 evaluated patients, 17 (12 %) had status = “No” for *VHL* inactivation. The remaining 20 (2 %) had status = “incomplete” (partial assay success) or “fail” (e.g., no DNA).

The average age, weight, height, and race distribution were similar between the two treatment arms (Supplemental Table 3). However, as noted for the IHC results, the performance and risk group status at baseline showed an imbalance between the two arms. The four VHL molecular markers summarized were overall *VHL* inactivation status, *VHL* mutation status, *VHL* large deletion status, and *VHL* methylation status.

For the combined arm analysis, TTP, PFS, and OS curves were analyzed separately, comparing the “yes” and “no” status of patients for each of the *VHL* inactivation groups (overall, mutation, deletion, and methylation); no statistically significant differences (adjusted or unadjusted) were observed (data not shown). The summary statistics for the VHL molecular markers were produced for each objective tumor response category (Table 4).

**Table 4:** Summary of *VHL* gene inactivation mechanism by best overall response (RECIST v1.0) in patients from both treatment arms combined

	No. of patients (%)			
	CR (*n* = 1)	PR (*n* = 51)	SD (*n* = 64)	PD (*n* = 23)
Overall *VHL* gene inactivation status				
Yes	0	41 (80)	45 (70)	18 (78)
No	0	5 (10)	10 (16)	2 (9)
*VHL* mutation inactivation status				
Yes	0	36 (71)	40 (62)	14 (61)
No	1 (100)	12 (24)	18 (28)	7 (30)
*VHL* large deletion status				
Yes	0	4 (8)	1 (2)	1 (4)
No	0	41 (80)	50 (78)	18 (78)
*VHL* methylation status				
Yes	0	5 (10)	5 (8)	4 (17)
No	1 (100)	45 (88)	54 (84)	18 (78)

Overall *VHL* gene inactivation status could = no, yes, fail, or incomplete. The status = “yes” if at least one *VHL* status (mutational, methylation, or deletion) = “yes”; = “fail” if *VHL* mutational status was of unknown significance; = “no” if all three were “no”; and = “incomplete” if all tests were not successful

*CR* Complete response, *PD* progressive disease, *PR* partial response, *RECIST* response evaluation criteria in solid tumors, *SD* stable disease, *VHL* Von Hippel–Lindau

In the analyses of Schedule 4/2 patients only, a trend for significance (*p* = 0.076) was observed for a comparison of TTP and PFS curves based on stratification by VHL mutation inactivation status. Patients whose status was “yes,” compared with patients whose status was “no,” had longer survival [hazard ratio (HR), 0.56; 95 % confidence interval (CI), 0.30–1.07], with results identical for both unadjusted analyses. The adjusted *p* value comparisons were not statistically significant.

## Discussion

These analyses provide evidence that serum Ang-2 and MMP-2, and tumor HIF-1α are potential baseline efficacy biomarkers for sunitinib in advanced RCC (based on unadjusted *p* value comparisons to *α* = 0.05) that warrant further analysis, including further understanding of their prognostic versus predictive value.

The two multiplex platforms for soluble proteins are utilizing very different methodologies, and although the two platforms shared 36 overlapping proteins, only the two baseline biomarkers that correlated with response on the SearchLight platform, low Ang-2 and high MMP-2, were also identified in the protein signatures of approximately 40 markers associated with best overall response, or response extreme, on the SOMAscan platform. Technical differences in assay platforms and their performances likely contribute to the absence of additional proteins identified by the two platforms. Interestingly, the Ang-2/Tie-2 axis has been identified as a potential resistance mechanism to RTK inhibition by engaging a parallel pro-angiogenic pathway [22]. A phase II study of an Ang-2 inhibitor (PF-04856884), in combination with the VEGFR1, 2, and 3 inhibitor axitinib, is testing the hypothesis that joint inhibition of the compensatory Ang-2 and VEGFR pathways may offer superior efficacy to axitinib alone (ClinicalTrials.gov: NCT01441414). Another potential resistance mechanism and escape from antiangiogenic therapy involves the HGF/c-Met axis [22, 23]; while lower HGF baseline levels were associated with response on the SearchLight platform, these were not identified on the SOMAscan platform. However, on both platforms, FDRs were high, indicating the associations are weak and unreliable and, therefore, unlikely to be useful for patient selection. Separation of prognostic versus predictive value for sunitinib therapy specifically is not established. Furthermore, prior findings, such as association of lower baseline levels of sVEGFR3 and VEGF-C with longer PFS and ORR in sunitinib-treated patients with bevacizumab-refractory mRCC, were not replicated here [3].

In addition, results of baseline soluble protein studies for other VEGFR2 inhibitors appear to differ from those reported here for sunitinib. For example, in a large retrospective analysis of studies with pazopanib [5], using data from mRCC patients in a phase II trial (*n* = 215) and a randomized, placebo-controlled phase III trial (*n* = 344), the following seven cytokine and angiogenic factors (CAFs) were observed to be associated with either continuous tumor shrinkage or PFS with pazopanib out of a panel of 17 CAFs: IL-6, IL-8, VEGF, osteopontin, E-selectin, HGF, and TIMP-1 [5]. In a retrospective study of sorafenib [6], using data from mRCC patients in a randomized phase II trial of sorafenib with or without interferon-α (*n* = 69), the following six-baseline CAF signature was found to be correlated with PFS benefit with sorafenib out of a panel of 52 CAFs: osteopontin, VEGF, CA-IX, collagen IV, VEGFR2, and tumor necrosis factor-related apoptosis-inducing ligand (TRAIL).

For the analysis of serum soluble protein modulations (end of treatment compared with baseline, which was limited to 26 paired samples), there was limited convergence between the two platforms. On the SearchLight platform, six soluble proteins were statistically significantly modulated and corresponded to FDR ≤ 0.05: G-CSF; CRP; IL-6; MMP-1; PlGF; and SCF. All six increased following sunitinib treatment and included markers of inflammation (CRP); recruitment/activation of monocytes (G-CSF and IL-6); angiogenic signaling (PlGF); and pharmacodynamic targeting of sunitinib (SCF). On the SOMAscan platform, 15 markers were modulated following sunitinib treatment and were highly statistically significant (both unadjusted and adjusted for repeat analyses), with an associated FDR < 0.0026: CDK8/cyclinC; FST; Factor I; IL-16; IL-31; IR; IgM; Karyopherin-a2; MMP-2; NovH; SCF sR; TFPI; TPSB2; VEGF sR2; and sL-Selectin. Similar to the SearchLight signature, this includes markers of inflammation, recruitment/activation of monocytes/lymphocytes, and pharmacodynamic targets of sunitinib, e.g., sKIT and sVEGFR2. However, none of the proteins associated with those signatures remained statistically significantly modulated in the best overall response and extreme response categories, with the exception of SCF in the SearchLight platform. Sunitinib is a potent inhibitor of KIT, the receptor for SCF, and blockade of this pathway may lead to overexpression to counteract this inhibitory effect. Interestingly, in a recent study of advanced RCC patients (*n* = 85), focused on measuring circulating levels of three pro-angiogenic cytokines (IL-6, bFGF, and HGF), progression on sunitinib was preceded by increases in circulating levels of all three markers [24].

While genotyping in this study involved germ line DNA (blood) rather than tumor tissue, the approach was considered valid, since it is a host (as opposed to tumor)-regulated process. In addition, in a previous study of breast cancer patients, there was 100 % concordance between VEGF pathway-related SNPs in primary breast tumor and germ line DNA [25].

To date, SNPs in *VEGF*-*A* (*VEGF*-*A*-*2578*, *rs699947*; and *VEGF*-*A*-*1154*, *rs1570360*) have been associated with OS for advanced breast cancer patients receiving the anti-VEGF monoclonal antibody bevacizumab in combination with standard-of-care chemotherapy options (paclitaxel) [8]. SNPs in the *VEGF*-*A* gene have also been associated with efficacy for mRCC patients receiving axitinib [7], whereas SNPs in the *VEGFR3* gene have been associated with sunitinib efficacy [10].

In the current study, there were no statistically significant correlations at the 0.05 level between any of the selected *VEGF*-*A* or *VEGFR3* SNPs and TTP, PFS, OS or ORR when the two treatment arms were combined. The absence of statistically significant correlations suggests that there is an absence of a strong influence of the selected SNPs on RCC patient prognosis. The current study recruited more patients than, or at least approximately equivalent to, prior studies, yet did not replicate previous findings, in which, for example, two *VEGFR3* SNPs (*rs307821* and *rs307826*) were associated with reduced PFS to sunitinib in advanced RCC patients [10]. The absence of replication suggests weak or no influence of the selected *VEGF*-*A* or *VEGFR3* SNPs on RCC patient outcome.

Hypoxia-inducible factor 1-alpha (HIF-1α) and CA-IX are hypoxia tumor markers. For the combined treatment arm analyses of IHC tumor biomarkers, there were no statistically significant associations between TTP and OS with either HIF-1α or CA-IX percent of tumor expression; however, in the analysis of HIF-1α percent of tumor expression, PFS was statistically significantly different between groups 0–2 (i.e., 0–50 % or low expression) versus groups 3–4 (i.e., 51–100 % or high expression) before multiple testing. The absence of statistical significance after multiple testing adjustment indicates potential weakness in the association. This finding is broadly consistent with the results from a similar study of 67 mRCC patients who had received antiangiogenic therapies [sunitinib (*n* = 61), including as first-line treatment (*n* = 60); sorafenib (*n* = 21); bevacizumab (*n* = 3); or temsirolimus (*n* = 19)]; for sunitinib-treated patients, lower HIF-1α expression was associated with statistically significantly longer PFS [11], possibly due to lesser angiogenesis pathway activation. While the statistical methodology of assessing HIF-1α expression was different compared with the current investigation, the overall results are consistent.

For the combined treatment arm analysis of biomarkers based on *VHL* inactivation status, no statistically significant differences in the TTP, PFS, or OS curves were observed (adjusted or unadjusted); however, there was a trend for longer TTP and PFS (unadjusted) in Schedule 4/2 patients whose inactivation status was “yes.” To provide context for these data, a previous study of *VHL* mutation status in 134 RCC patients who received sorafenib as part of the Treatment Approaches in Renal Cancer Global Evaluation Trial (TARGET) showed no association between *VHL* mutation status and clinical benefit [12]. The major differences with the current study were (1) only mutation status was assessed in TARGET, not methylation or copy number status, and (2) sorafenib has a different pharmacologic profile than sunitinib. Another study of *VHL* inactivation status in 123 RCC patients who received either sunitinib, sorafenib, axitinib, or bevacizumab found no statistically significant increase in response to these VEGF-targeted agents [26]. All three studies report similar outcomes with regard to the absence of a strong predictive value of *VHL* mutation status with respect to outcome in advanced RCC patients receiving agents disrupting the VEGF/VEGFR2 pathway.

In conclusion, our findings relate to the activity of and potential resistance mechanisms to sunitinib in advanced RCC. However, clinical utility of serum Ang-2 and MMP-2 and tumor HIF-1α was not addressed at this time and would need to be further evaluated and demonstrated. Separation of predictive versus prognostic value cannot be made for this study in the absence of a placebo arm. Finally, the existence of several indirect resistance mechanisms for antiangiogenic agents [27] in advanced RCC [22] provides significant challenge for the identification and validation of patient selection tests for sunitinib treatment.

## Electronic supplementary material

Below is the link to the electronic supplementary material.

Supplementary material 1 (DOC 1100 kb)
