# Supplementary material for: Investigation of novel circulating proteins, germ line single-nucleotide polymorphisms, and molecular tumor markers as potential efficacy biomarkers of first-line sunitinib therapy for advanced renal cell carcinoma
Source: Cancer Chemother Pharmacol. 2014 Aug 7;74(4):739–50. doi: 10.1007/s00280-014-2539-0 (PMC4175044; doi:10.1007/s00280-014-2539-0)
Supplement: Supplementary file 1 — Supplementary material 1 (DOC 1100 kb) [file 280_2014_2539_MOESM1_ESM.doc]

**Supplemental tables**

**Supplemental Table 1 Menu of analytes covered by the SomaLogic SOMAscan proteomic multiplex platform**

| Protein name | Entrez gene |
| --- | --- |
| C4b | *C4A C4B* |
| Coagulation factor XI | *F11* |
| CTACK | *CCL27* |
| Endostatin | *COL18A1* |
| TIMP-1 | *TIMP1* |
| tPA | *PLAT* |
| EG-VEGF | *PROK1* |
| TIMP-2 | *TIMP2* |
| SDF-1a | *CXCL12* |
| TGF-b1 | *TGFB1* |
| VEGF sR3 | *FLT4* |
| C5 | *C5* |
| Apo E | *APOE* |
| BDNF | *BDNF* |
| bFGF-R | *FGFR1* |
| C8 | *C8A C8B C8G* |
| Cathepsin G | *CTSG* |
| CXCL16, soluble | *CXCL16* |
| FGF-8B | *FGF8* |
| GIIE | *PLA2G2E* |
| GV | *PLA2G5* |
| MIP-3a | *CCL20* |
| SAP | *APCS* |
| SCF sR | *KIT* |
| TIMP-3 | *TIMP3* |
| Tpo | *THPO* |
| Troponin I | *TNNI3* |
| Cadherin E | *CDH1* |
| Ephrin-B3 | *EFNB3* |
| GFRa-2 | *GFRA2* |
| 6Ckine | *CCL21* |
| RANTES | *CCL5* |
| HMG-1 | *HMGB1* |
| b-Endorphin | *POMC* |
| Factor I | *CFI* |
| IGFBP-2 | *IGFBP2* |
| IGFBP-3 | *IGFBP3* |
| Leptin | *LEP* |
| MCP-1 | *CCL2* |
| MMP-9 | *MMP9* |
| Myeloperoxidase | *MPO* |
| PRL | *PRL* |
| PSA | *KLK3* |
| ROR1 | *ROR1* |
| VEGF | *VEGFA* |
| 4-1BB | *TNFRSF9* |
| 4-1BB ligand | *TNFSF9* |
| Angiopoietin-2 | *ANGPT2* |
| B7 | *CD80* |
| CD30 | *TNFRSF8* |
| CLF-1/CLC Complex | *CRLF1 CLCF1* |
| Cystatin C | *CST3* |
| Dtk | *TYRO3* |
| eIF-5 | *EIF5* |
| Ephrin-A4 | *EFNA4* |
| Ephrin-A5 | *EFNA5* |
| ERBB2 | *ERBB2* |
| ERBB3 | *ERBB3* |
| ERBB4 | *ERBB4* |
| GA733-1 protein | *TACSTD2* |
| gp130, soluble | *IL6ST* |
| HO-2 | *HMOX2* |
| HPV E7 Type18 | *Human-virus* |
| HSP 90a | *HSP90AA1* |
| HSP 90b | *HSP90AB1* |
| IL-1 R AcP | *IL1RAP* |
| IL-10 Rb | *IL10RB* |
| IL-13 Ra1 | *IL13RA1* |
| IL-2 sRg | *IL2RG* |
| Layilin | *LAYN* |
| Lymphotoxin b R | *LTBR* |
| Macrophage mannose receptor | *MRC1* |
| M-CSF R | *CSF1R* |
| MSP R | *MST1R* |
| PAFAH beta subunit | *PAFAH1B2* |
| P-Cadherin | *CDH3* |
| PKC-A | *PRKCA* |
| PKC-Z | *PRKCZ* |
| Rab GDP dissociation inhibitor beta | *GDI2* |
| suPAR | *PLAUR* |
| TNF sR-I | *TNFRSF1A* |
| TrkC | *NTRK3* |
| BCMA | *TNFRSF17* |
| Bone proteoglycan II | *DCN* |
| Calpain I | *CAPN1 CAPNS1* |
| CK-MM | *CKM* |
| Cripto | *TDGF1* |
| ERBB1 | *EGFR* |
| HGF | *HGF* |
| HSP 60 | *HSPD1* |
| iC3b | *C3* |
| IGFBP-5 | *IGFBP5* |
| IGFBP-6 | *IGFBP6* |
| MIA | *MIA* |
| NEUREGULIN-1 | *NRG1* |
| NPS-PLA2 | *PLA2G2A* |
| OSM | *OSM* |
| PECAM-1 | *PECAM1* |
| Persephin | *PSPN* |
| PF-4 | *PF4* |
| Protein S | *PROS1* |
| TECK | *CCL25* |
| Thyroxine-Binding Globulin | *SERPINA7* |
| TNFSF18 | *TNFSF18* |
| CNTFR alpha | *CNTFR* |
| EMAP-2 | *AIMP1* |
| EPO-R | *EPOR* |
| WISP-3 | *WISP3* |
| IL-1F7 | *IL1F7* |
| IL-7 Ra | *IL7R* |
| Laminin | *LAMA1 LAMB1 LAMC1* |
| MICA | *MICA* |
| NADPH-P450 Oxidoreductase | *POR* |
| NANOG | *NANOG* |
| NKp44 | *NCR2* |
| Noggin | *NOG* |
| NovH | *NOV* |
| RELT | *RELT* |
| Siglec-6 | *SIGLEC6* |
| Siglec-7 | *SIGLEC7* |
| Sonic Hedgehog | *SHH* |
| IgG | *IGHG1 IGHG2 IGHG3 IGHG4* |
| TSLP R | *CRLF2* |
| ULBP-3 | *ULBP3* |
| Activin A | *INHBA* |
| Apo A-I | *APOA1* |
| Azurocidin | *AZU1* |
| BMP-14 | *GDF5* |
| C1q | *C1QA C1QB C1QC* |
| C3 | *C3* |
| C3adesArg | *C3* |
| DRR1 | *FAM107A* |
| FGF-18 | *FGF18* |
| FGF-19 | *FGF19* |
| FGF-20 | *FGF20* |
| FGF9 | *FGF9* |
| GDF-11 | *GDF11* |
| Hemopexin | *HPX* |
| HIV-2 Rev | *Human-virus* |
| I-309 | *CCL1* |
| IGFBP-1 | *IGFBP1* |
| IL-16 | *IL16* |
| IL-17F | *IL17F* |
| IL-22 | *IL22* |
| Lactoferrin | *LTF* |
| LAG-1 | *CCL4L1* |
| LD78-beta | *CCL3L1* |
| MCP-2 | *CCL8* |
| MMP-7 | *MMP7* |
| NAP-2 | *PPBP* |
| SOD | *SOD1* |
| Alkaline phosphatase, bone | *ALPL* |
| Fibrinogen | *FGA FGB FGG* |
| Apo B | *APOB* |
| ACE2 | *ACE2* |
| Activin RIB | *ACVR1B* |
| ADAMTS-4 | *ADAMTS4* |
| Angiopoietin-1 | *ANGPT1* |
| ART | *AGRP* |
| BCAM | *BCAM* |
| Cadherin-5 | *CDH5* |
| CD97 | *CD97* |
| COMMD7 | *COMMD7* |
| EDA | *EDA* |
| Fractalkine/CX3CL-1 | *CX3CL1* |
| HAI-1 | *SPINT1* |
| IL-27 | *IL27* |
| Kallikrein 11 | *KLK11* |
| Kallikrein 4 | *KLK4* |
| kallikrein 8 | *KLK8* |
| Ku70 | *XRCC6* |
| Lipocalin 2 | *LCN2* |
| Met | *MET* |
| OX40 Ligand | *TNFSF4* |
| sFRP-3 | *FRZB* |
| sICAM-2 | *ICAM2* |
| SPINT2 | *SPINT2* |
| sTie-1 | *TIE1* |
| Ubiquitin+1 | *RPS27A* |
| WIF-1 | *WIF1* |
| AIF1 | *AIF1* |
| C5a | *C5* |
| CHK1 | *CHEK1* |
| ERK-1 | *MAPK3* |
| Glucocorticoid receptor | *NR3C1* |
| Hat1 | *HAT1* |
| HDAC8 | *HDAC8* |
| Karyopherin-a2 | *KPNA2* |
| MOZ | *MYST3* |
| PKB | *AKT1* |
| PKC-B-II | *PRKCB* |
| RAC1 | *RAC1* |
| TBP | *TBP* |
| Topoisomerase I | *TOP1* |
| UBC9 | *UBE2I* |
| YES | *YES1* |
| a1-Antichymotrypsin | *SERPINA3* |
| C7 | *C7* |
| Cardiotrophin-1 | *CTF1* |
| CCL28 | *CCL28* |
| CD22 | *CD22* |
| HCC-1 | *CCL14* |
| IL-4 | *IL4* |
| Midkine | *MDK* |
| MPIF-1 | *CCL23* |
| NAP-2 | *PPBP* |
| PCNA | *PCNA* |
| sRANKL | *TNFSF11* |
| PAI-1 | *SERPINE1* |
| Apo E3 | *APOE* |
| Apo E4 | *APOE* |
| Artemin | *ARTN* |
| Cytochrome c | *CYCS* |
| Cytochrome P450 3A4 | *CYP3A4* |
| DAN | *NBL1* |
| ER | *ESR1* |
| Factor D | *CFD* |
| GX | *PLA2G10* |
| IGFBP-4 | *IGFBP4* |
| IGF-I | *IGF1* |
| Luteinizing hormone | *CGA LHB* |
| MMP-8 | *MMP8* |
| NG36 | *EHMT2* |
| Properdin | *CFP* |
| Protein C | *PROC* |
| PTHrP | *PTHLH* |
| SCGF-beta | *CLEC11A* |
| VCAM-1 | *VCAM1* |
| TNFSF15 | *TNFSF15* |
| ALK-1 | *ACVRL1* |
| AREG | *AREG* |
| BMP-7 | *BMP7* |
| CD36 ANTIGEN | *CD36* |
| contactin-1 | *CNTN1* |
| Desmoglein-1 | *DSG1* |
| EDAR | *EDAR* |
| ENA-78 | *CXCL5* |
| ESAM | *ESAM* |
| Galectin-4 | *LGALS4* |
| Gro-a | *CXCL1* |
| Gro-g | *CXCL3* |
| Histone H1.2 | *HIST1H1C* |
| ICOS | *ICOS* |
| IL-1 sRI | *IL1R1* |
| IL-17 sR | *IL17RA* |
| IL-18 Rb | *IL18RAP* |
| IL-1Rrp2 | *IL1RL2* |
| JAM-B | *JAM2* |
| JAM-C | *JAM3* |
| LSAMP | *LSAMP* |
| MBL | *MBL2* |
| NKp30 | *NCR3* |
| PTP-1B | *PTPN1* |
| Siglec-9 | *SIGLEC9* |
| TGF-b R III | *TGFBR3* |
| TSLP | *TSLP* |
| CTLA-4 | *CTLA4* |
| a2-Antiplasmin | *SERPINF2* |
| bFGF | *FGF2* |
| Calpastatin | *CAST* |
| Ck-b-8-1 | *CCL23* |
| DC-SIGN | *CD209* |
| DC-SIGNR | *CLEC4M* |
| Ferritin | *FTH1 FTL* |
| FSH | *CGA FSHB* |
| Galectin-2 | *LGALS2* |
| GFAP | *GFAP* |
| IL-19 | *IL19* |
| I-TAC | *CXCL11* |
| MIP-1a | *CCL3* |
| MRC2 | *MRC2* |
| Myoglobin | *MB* |
| ON | *SPARC* |
| PARC | *CCL18* |
| PTN | *PTN* |
| resistin | *RETN* |
| Trypsin | *PRSS1* |
| vWF | *VWF* |
| Fas ligand, soluble | *FASLG* |
| Flt3 ligand | *FLT3LG* |
| Haptoglobin, Mixed Type | *HP* |
| IL-4 sR | *IL4R* |
| NKG2D | *KLRK1* |
| WISP-1 | *WISP1* |
| BAFF | *TNFSF13B* |
| C9 | *C9* |
| Cathepsin B | *CTSB* |
| FGF-5 | *FGF5* |
| Galectin-3 | *LGALS3* |
| GDF-9 | *GDF9* |
| IgM | *IGHM* |
| IL-2 | *IL2* |
| IL-13 | *IL13* |
| IL-18 BPa | *IL18BP* |
| LBP | *LBP* |
| Coagulation Factor Xa | *F10* |
| PlGF | *PGF* |
| TIG2 | *RARRES2* |
| ULBP-1 | *ULBP1* |
| ULBP-2 | *ULBP2* |
| XEDAR | *EDA2R* |
| Aurora kinase A | *AURKA* |
| DARPP-32 | *PPP1R1B* |
| DEAD-box protein 19B | *DDX19B* |
| SMAC | *DIABLO* |
| TRAIL R4 | *TNFRSF10D* |
| VEGF-C | *VEGFC* |
| Gro-b | *CXCL2* |
| IL-2 sRa | *IL2RA* |
| TNF sR-II | *TNFRSF1B* |
| Siglec-3 | *CD33* |
| ADAMTS-5 | *ADAMTS5* |
| IDUA | *IDUA* |
| AMPM2 | *METAP2* |
| amyloid precursor protein | *APP* |
| ARSB | *ARSB* |
| ASAHL | *NAAA* |
| ATS1 | *ADAMTS1* |
| ATS13 | *ADAMTS13* |
| Carbonic Anhydrase IV | *CA4* |
| CATC | *CTSC* |
| Cathepsin A | *CTSA* |
| Cathepsin D | *CTSD* |
| Cathepsin S | *CTSS* |
| CD39 | *ENTPD1* |
| Coagulation Factor VII | *F7* |
| C2 | *C2* |
| CRIS3 | *CRISP3* |
| Enterokinase | *PRSS7* |
| GAS1 | *GAS1* |
| Glutamate carboxypeptidase | *CNDP2* |
| GPVI | *GP6* |
| Granulysin | *GNLY* |
| HPLN1 | *HAPLN1* |
| IDE | *IDE* |
| IDS | *IDS* |
| kallikrein 12 | *KLK12* |
| kallikrein 5 | *KLK5* |
| KREM2 | *KREMEN2* |
| LKHA4 | *LTA4H* |
| LYVE1 | *LYVE1* |
| MATN3 | *MATN3* |
| MEPE | *MEPE* |
| METAP1 | *METAP1* |
| ASAH2 | *ASAH2* |
| Nidogen | *NID1* |
| NRP1 | *NRP1* |
| PIGR | *PIGR* |
| Protease nexin I | *SERPINE2* |
| RET | *RET* |
| SARP-2 | *SFRP1* |
| Semaphorin 3A | *SEMA3A* |
| TrATPase | *ACP5* |
| URB | *CCDC80* |
| GSK-3 beta | *GSK3B* |
| Aggrecan | *ACAN* |
| ANGL3 | *ANGPTL3* |
| BGH3 | *TGFBI* |
| BGN | *BGN* |
| C1r | *C1R* |
| Carbonic Anhydrase X | *CA10* |
| CD109 | *CD109* |
| CD23 | *FCER2* |
| CD48 | *CD48* |
| CD5L | *CD5L* |
| CNTN2 | *CNTN2* |
| Contactin-4 | *CNTN4* |
| Contactin-5 | *CNTN5* |
| CYTF | *CST7* |
| Cystatin M | *CST6* |
| CYTN | *CST1* |
| DLL4 | *DLL4* |
| FCG2A | *FCGR2A* |
| FCG2B | *FCGR2B* |
| FCG3B | *FCGR3B* |
| FCGR1 | *FCGR1A* |
| GFRa-1 | *GFRA1* |
| GPC2 | *GPC2* |
| Heparin cofactor II | *SERPIND1* |
| IGFBP-7 | *IGFBP7* |
| IL-24 | *IL24* |
| LRIG3 | *LRIG3* |
| LRP8 | *LRP8* |
| LY9 | *LY9* |
| MATN2 | *MATN2* |
| Nectin-like protein 2 | *CADM1* |
| NET4 | *NTN4* |
| PGRP-S | *PGLYRP1* |
| RGMB | *RGMB* |
| RGM-C | *HFE2* |
| Testican-2 | *SPOCK2* |
| TFPI | *TFPI* |
| TSP2 | *THBS2* |
| TSP4 | *THBS4* |
| Aminoacylase-1 | *ACY1* |
| Antithrombin III | *SERPINC1* |
| AURKB | *AURKB* |
| BARK1 | *ADRBK1* |
| BMP-1 | *BMP1* |
| CAMK2A | *CAMK2A* |
| Carbonic anhydrase 6 | *CA6* |
| Carbonic anhydrase VII | *CA7* |
| CDK2/cyclin A | *CDK2 CCNA2* |
| CDK5/p35 | *CDK5 CDK5R1* |
| CDK8/cyclin C | *CDK8 CCNC* |
| Chk2 | *CHEK2* |
| CLC4K | *CD207* |
| CRDL1 | *CHRDL1* |
| CSK | *CSK* |
| Cathepsin V | *CTSL2* |
| Dkk-4 | *DKK4* |
| ECM1 | *ECM1* |
| FETUB | *FETUB* |
| Granzyme H | *GZMH* |
| HCK | *HCK* |
| Kallikrein 7 | *KLK7* |
| KPCI | *PRKCI* |
| LYNB | *LYN* |
| PCI | *SERPINA5* |
| PIK3CA/PIK3R1 | *PIK3CA PIK3R1* |
| PK3CG | *PIK3CG* |
| PKB gamma | *AKT3* |
| Renin | *REN* |
| SHP-2 | *PTPN11* |
| STAB2 | *STAB2* |
| TBK1 | *TBK1* |
| TCPTP | *PTPN2* |
| TPSB2 | *TPSB2* |
| TPSG1 | *TPSG1* |
| UFC1 | *UFC1* |
| WNK3 | *WNK3* |
| ALCAM | *ALCAM* |
| Bcl-2 | *BCL2* |
| BFL1 | *BCL2A1* |
| BMX | *BMX* |
| BSP | *IBSP* |
| BTK | *BTK* |
| CAMK1D | *CAMK1D* |
| CAMK2D | *CAMK2D* |
| Carbonic anhydrase XIII | *CA13* |
| CD30 Ligand | *TNFSF8* |
| CDK1/cyclin B | *CDC2 CCNB1* |
| Chymase | *CMA1* |
| EphA1 | *EPHA1* |
| EPHA3 | *EPHA3* |
| FN1.3 | *FN1* |
| FN1.4 | *FN1* |
| Flt-3 | *FLT3* |
| FSTL3 | *FSTL3* |
| granzyme A | *GZMA* |
| GSK-3 alpha | *GSK3A* |
| HIPK3 | *HIPK3* |
| IL-15 Ra | *IL15RA* |
| IL-18 Ra | *IL18R1* |
| IL-8 | *IL8* |
| IR | *INSR* |
| Kallistatin | *SERPINA4* |
| Kallikrein 6 | *KLK6* |
| LCK | *LCK* |
| LYN | *LYN* |
| Osteoblast-specif transcr fact 2 | *RUNX2* |
| PDGF Rb | *PDGFRB* |
| PGCB | *BCAN* |
| PRKACA | *PRKACA* |
| RPS6KA3 | *RPS6KA3* |
| sE-Selectin | *SELE* |
| STK16 | *STK16* |
| Survivin | *BIRC5* |
| Thrombopoietin Receptor | *MPL* |
| Thrombospondin-1 | *THBS1* |
| TRY3 | *PRSS3* |
| DUS3 | *DUSP3* |
| XPNPEP1 | *XPNPEP1* |
| Angiotensinogen | *AGT* |
| b2-Microglobulin | *B2M* |
| b-ECGF | *FGF1* |
| BLC | *CXCL13* |
| Catalase | *CAT* |
| Epo | *EPO* |
| GCP-2 | *CXCL6* |
| IL-17 | *IL17A* |
| IL-17B | *IL17B* |
| Integrin a1b1 | *ITGA1 ITGB1* |
| LEAP-1 | *HAMP* |
| Lymphotoxin a1/b2 | *LTA LTB* |
| MDC | *CCL22* |
| MIP-5 | *CCL15* |
| Proteinase-3 | *PRTN3* |
| SDF-1b | *CXCL12* |
| TAFI | *CPB2* |
| TARC | *CCL17* |
| TGF-b3 | *TGFB3* |
| TSH | *CGA TSHB* |
| CD40 ligand, soluble | *CD40LG* |
| DKK1 | *DKK1* |
| dopa decarboxylase | *DDC* |
| Adiponectin | *ADIPOQ* |
| a1-Antitrypsin | *SERPINA1* |
| a2-HS-Glycoprotein | *AHSG* |
| Arylsulfatase A | *ARSA* |
| BASI | *BSG* |
| BMP10 | *BMP10* |
| C1s | *C1S* |
| Cadherin-6 | *CDH6* |
| CAMK1 | *CAMK1* |
| Caspase-3 | *CASP3* |
| CATE | *CTSE* |
| CEA | *CEACAM5* |
| Chitotriosidase-1 | *CHIT1* |
| CHL1 | *CHL1* |
| CLC7A | *CLEC7A* |
| CNDP1 | *CNDP1* |
| MASP3 | *MASP1* |
| Discoidin domain receptor 2 | *DDR2* |
| DKK3 | *DKK3* |
| DPP2 | *DPP7* |
| Endothelin-converting enzyme 1 | *ECE1* |
| EphB4 | *EPHB4* |
| FCN1 | *FCN1* |
| GNS | *GNS* |
| HGFA | *HGFAC* |
| IL22RA1 | *IL22RA1* |
| LGMN | *LGMN* |
| LY86 | *LY86* |
| Marapsin | *PRSS27* |
| MMEL2 | *MMEL1* |
| MP2K2 | *MAP2K2* |
| MRCKB | *CDC42BPB* |
| Nectin-like protein 1 | *CADM3* |
| NID2 | *NID2* |
| OBCAM | *OPCML* |
| OCAD1 | *OCIAD1* |
| OLR1 | *OLR1* |
| PKC-G | *PRKCG* |
| RAP | *LRPAP1* |
| RBP | *RBP4* |
| SLAF5 | *CD84* |
| SLIK1 | *SLITRK1* |
| Soggy-1 | *DKKL1* |
| TEC | *TEC* |
| WFKN2 | *WFIKKN2* |
| VEGF sR2 | *KDR* |
| BMPER | *BMPER* |
| Cadherin-12 | *CDH12* |
| Calcineurin B a | *PPP3R1* |
| COLEC12 | *COLEC12* |
| complement factor H-related 5 | *CFHR5* |
| IL-17 RC | *IL17RC* |
| IGF-II receptor | *IGF2R* |
| kallikrein 14 | *KLK14* |
| Macrophage scavenger receptor | *MSR1* |
| MFRP | *MFRP* |
| Testican-1 | *SPOCK1* |
| IgG | *IGHG1 IGHG2 IGHG3 IGHG4* |
| Albumin | *ALB* |
| a2-Macroglobulin | *A2M* |
| ALT | *GPT* |
| Angiostatin | *PLG* |
| BAFF Receptor | *TNFRSF13C* |
| CK-MB | *CKB CKM* |
| IFN-g R1 | *IFNGR1* |
| p27Kip1 | *CDKN1B* |
| TNF-a | *TNF* |
| Gastrin-releasing peptide | *GRP* |
| PTH | *PTH* |
| PYY | *PYY* |
| Secretin | *SCT* |
| Somatostatin-28 | *SST* |
| TNR4 | *TNFRSF4* |
| BMP-6 | *BMP6* |
| Cathepsin H | *CTSH* |
| CSF-1 | *CSF1* |
| gpIIbIIIa | *ITGA2B ITGB3* |
| IL-5 | *IL5* |
| MMP-10 | *MMP10* |
| CD70 | *CD70* |
| Activated Protein C | *PROC* |
| Activated Protein C | *PROC* |
| COX-2 | *PTGS2* |
| STX1a | *STX1A* |
| sTie-2 | *TEK* |
| ADAM 9 | *ADAM9* |
| ANGL4 | *ANGPTL4* |
| Cadherin-2 | *CDH2* |
| Carbonic anhydrase III | *CA3* |
| CK-BB | *CKB* |
| CSK21 | *CSNK2A1* |
| CYTD | *CST5* |
| DMP1 | *DMP1* |
| Endocan | *ESM1* |
| EphA5 | *EPHA5* |
| FGF23 | *FGF23* |
| FGFR-2 | *FGFR2* |
| FGFR-3 | *FGFR3* |
| FGR | *FGR* |
| Ficolin-3 | *FCN3* |
| FYN | *FYN* |
| IL-11 RA | *IL11RA* |
| IL-12 RB2 | *IL12RB2* |
| KPCT | *PRKCQ* |
| MAPK2 | *MAPKAPK2* |
| MAPKAPK3 | *MAPKAPK3* |
| MATK | *MATK* |
| MK08 | *MAPK8* |
| PAK6 | *PAK6* |
| PDGF-CC | *PDGFC* |
| pTEN | *PTEN* |
| PTK6 | *PTK6* |
| RGMA | *RGMA* |
| SRCN1 | *SRC* |
| TLR2 | *TLR2* |
| ZAP70 | *ZAP70* |
| 14-3-3 protein zeta/delta | *YWHAZ* |
| aldolase A | *ALDOA* |
| CN166 | *C14orf166* |
| Cyclophilin A | *PPIA* |
| DLRB1 | *DYNLRB1* |
| eIF-4H | *EIF4H* |
| ETHE1 | *ETHE1* |
| GAPDH, liver | *GAPDH* |
| HSP 40 | *DNAJB1* |
| MDHC | *MDH1* |
| NACA | *NACA* |
| Peroxiredoxin-1 | *PRDX1* |
| PPAC | *ACP1* |
| PSA1 | *PSMA1* |
| PSA6 | *PSMA6* |
| RACK1 | *GNB2L1* |
| RS3A | *RPS3A* |
| RS7 | *RPS7* |
| RSK-like protein kinase | *RPS6KA5* |
| SGTA | *SGTA* |
| Stress-induced-phosphoprotein 1 | *STIP1* |
| TCTP | *TPT1* |
| TMA | *TPO* |
| ARI3A | *ARID3A* |
| ASGR1 | *ASGR1* |
| CaMKK alpha | *CAMKK1* |
| CDC37 | *CDC37* |
| DLC8 | *DYNLL1* |
| HINT1 | *HINT1* |
| IMB1 | *KPNB1* |
| ING1 | *ING1* |
| LDH-H 1 | *LDHB* |
| MBD4 | *MBD4* |
| MED-1 | *MED1* |
| Mesothelin | *MSLN* |
| NAGK | *NAGK* |
| Phosphoglycerate mutase 1 | *PGAM1* |
| PLPP | *PDXP* |
| PSD7 | *PSMD7* |
| SAHH | *AHCY* |
| SKP1 | *SKP1* |
| Sorting nexin 4 | *SNX4* |
| UBE2N | *UBE2N* |
| discoidin domain receptor 1 | *DDR1* |
| FGF-4 | *FGF4* |
| HSP 70 | *HSPA1A* |
| sRAGE | *AGER* |
| BPI | *BPI* |
| C6 | *C6* |
| Eotaxin-2 | *CCL24* |
| Factor B | *CFB* |
| FGF-6 | *FGF6* |
| Fibronectin | *FN1* |
| FST | *FST* |
| Granzyme B | *GZMB* |
| IgE | *IGHE* |
| IL-17D | *IL17D* |
| IL-17E | *IL25* |
| IL-20 | *IL20* |
| IL-6 sRa | *IL6R* |
| IL-7 | *IL7* |
| IP-10 | *CXCL10* |
| Kininogen, HMW, Single Chain | *KNG1* |
| Lymphotactin | *XCL1* |
| MCP-4 | *CCL13* |
| Neurotrophin-5 | *NTF4* |
| PAPP-A | *PAPPA* |
| PDGF-BB | *PDGFB* |
| Plasmin | *PLG* |
| Plasminogen | *PLG* |
| Prekallikrein | *KLKB1* |
| PSA-ACT | *KLK3 SERPINA3* |
| P-Selectin | *SELP* |
| Tenascin | *TNC* |
| TGF-b2 | *TGFB2* |
| Thrombin | *F2* |
| uPA | *PLAU* |
| Factor H | *CFH* |
| MMP-2 | *MMP2* |
| Transferrin | *TF* |
| Histone H2A.z | *H2AFZ* |
| Thyroglobulin | *TG* |
| 14-3-3 protein gamma | *YWHAG* |
| 4EBP2 | *EIF4EBP2* |
| 6-Phosphogluconate dehydrogenase | *PGD* |
| Aflatoxin B1 aldehyde reductase | *AKR7A2* |
| AK1A1 | *AKR1A1* |
| BAD | *BAD* |
| Cofilin-1 | *CFL1* |
| DRG-1 | *VTA1* |
| Dynactin subunit 2 | *DCTN2* |
| EP15R | *EPS15L1* |
| ERAB | *HSD17B10* |
| Fascin | *FSCN1* |
| FER | *FER* |
| HNRPQ | *SYNCRIP* |
| IF4G2 | *EIF4G2* |
| IGF-I sR | *IGF1R* |
| IL-1 R4 | *IL1RL1* |
| LCMT1 | *LCMT1* |
| LIN7B | *LIN7B* |
| M2-PK | *PKM2* |
| MCM2 | *MCM2* |
| MDM2 | *MDM2* |
| NCAM-L1 | *L1CAM* |
| NDP kinase B | *NME2* |
| NSF1C | *NSFL1C* |
| NUDC3 | *NUDCD3* |
| OTUB1 | *OTUB1* |
| PA2G4 | *PA2G4* |
| paraoxonase 1 | *PON1* |
| Peroxiredoxin 2 | *PRDX2* |
| PESC | *PES1* |
| PEX5 | *PEX5* |
| PFD5 | *PFDN5* |
| PHI | *GPI* |
| prostatic binding protein | *PEBP1* |
| PSA2 | *PSMA2* |
| RAN | *RAN* |
| RBM39 | *RBM39* |
| SNAA | *NAPA* |
| Sphingosine kinase 1 | *SPHK1* |
| Spondin-1 | *SPON1* |
| Thymidine kinase | *TK1* |
| transcription factor MLR1, isoform CRA_b | *LCORL* |
| Transketolase | *TKT* |
| Triosephosphate isomerase | *TPI1* |
| XTP3A | *DCTPP1* |
| PTP-1C | *PTPN6* |
| AMNLS | *AMN* |
| CYTT | *CST2* |
| BOC | *BOC* |
| PSA | *KLK3* |
| CLC1B | *CLEC1B* |
| SAA | *SAA1* |
| CRP | *CRP* |
| sICAM-1 | *ICAM1* |
| DAPK2 | *DAPK2* |
| DYRK3 | *DYRK3* |
| Activin AB | *INHBA INHBB* |
| DHH | *DHH* |
| FGF-12 | *FGF12* |
| FGF-16 | *FGF16* |
| FGF-8A | *FGF8* |
| IFN-lambda 1 | *IL29* |
| IL-31 | *IL31* |
| SLPI | *SLPI* |
| SP-D | *SFTPD* |
| ADAM12 | *ADAM12* |
| BCL2-like 1 protein | *BCL2L1* |
| CHST2 | *CHST2* |
| Collectin Kidney 1 | *COLEC11* |
| ENPP7 | *ENPP7* |
| ENTP3 | *ENTPD3* |
| ENTP5 | *ENTPD5* |
| FCRL3 | *FCRL3* |
| GRB2-related adapter protein 2 | *GRAP2* |
| GREM1 | *GREM1* |
| hnRNP A/B | *HNRNPAB* |
| LRRT1 | *LRRTM1* |
| LRRT3 | *LRRTM3* |
| MFGM | *MFGE8* |
| MP2K4 | *MAP2K4* |
| PCSK7 | *PCSK7* |
| PDPK1 | *PDPK1* |
| RASA1 | *RASA1* |
| Sialoadhesin | *SIGLEC1* |
| SPARCL1 | *SPARCL1* |
| SPHK2 | *SPHK2* |
| ST4S6 | *CHST15* |
| TGM3 | *TGM3* |
| Tropomyosin 2 | *TPM2* |
| Ubiquitin | *RPS27A* |
| ZAP70 | *ZAP70* |
| ZAP70 | *ZAP70* |
| C1-Esterase Inhibitor | *SERPING1* |
| C3b | *C3* |
| C4 | *C4A C4B* |
| C5b, 6 Complex | *C5 C6* |
| DHH | *DHH* |
| FGF7 | *FGF7* |
| IL-3 Ra | *IL3RA* |
| IL-5 Ra | *IL5RA* |
| IL-11 | *IL11* |
| IL-23 | *IL12B IL23A* |
| Kininogen, HMW, Single Chain | *KNG1* |
| MMP-12 | *MMP12* |
| NCAM-120 | *NCAM1* |
| PDGF-AA | *PDGFA* |
| SCGF-alpha | *CLEC11A* |
| BSSP4 | *PRSS22* |
| BST1 | *BST1* |
| CBX5 | *CBX5* |
| CDON | *CDON* |
| Clusterin | *CLU* |
| CONA1 | *COL23A1* |
| CTAP-III | *PPBP* |
| EMR2 | *EMR2* |
| FLRT1 | *FLRT1* |
| Fucosyltransferase 3 | *FUT3* |
| FUT5 | *FUT5* |
| FYN | *FYN* |
| GP114 | *GPR114* |
| H6ST1 | *HS6ST1* |
| HDGR2 | *HDGFRP2* |
| IL-34 | *IL34* |
| KIRR3 | *KIRREL3* |
| KYNU | *KYNU* |
| LCK | *LCK* |
| Livin B | *BIRC7* |
| NXPH1 | *NXPH1* |
| PLCG1 | *PLCG1* |
| PLXC1 | *PLXNC1* |
| RSPO2 | *RSPO2* |
| SH21A | *SH2D1A* |
| SLIK5 | *SLITRK5* |
| SORC2 | *SORCS2* |
| PH | *PPY* |
| PACAP-27 | *ADCYAP1* |
| PACAP-38 | *ADCYAP1* |
| 3HIDH | *HIBADH* |
| FABP | *FABP3* |
| GM-CSF | *CSF2* |
| TNF-b | *LTA* |
| 41 | *EPB41* |
| 14-3-3 eta | *YWHAH* |
| 17-beta-HSD 1 | *HSD17B1* |
| 2B11 | *HLA-DRB1* |
| 3HAO | *HAAO* |
| Apo D | *APOD* |
| IL-3 | *IL3* |
| Protein disulfide isomerase A3 | *PDIA3* |
| TFF3 | *TFF3* |
| Afamin | *AFM* |
| Olfactomedin-4 | *OLFM4* |
| ASM3A | *SMPDL3A* |
| FAM107B | *FAM107B* |
| Gelsolin | *GSN* |
| CBG | *SERPINA6* |
| Cytidylate kinase | *CMPK1* |
| C34 gp41 HIV Fragment | *Human-virus* |
| PERL | *LPO* |
| CO8A1 | *COL8A1* |
| ITI heavy chain H4 | *ITIH4* |
| TXD12 | *TXNDC12* |
| sL-Selectin | *SELL* |
| TRAIL R1 | *TNFRSF10A* |
| Epithelial cell kinase | *EPHA2* |
| G-CSF | *CSF3* |
| Glypican 3 | *GPC3* |
| IL-1a | *IL1A* |
| BMPR1A | *BMPR1A* |
| BMP RII | *BMPR2* |
| TrkB | *NTRK2* |
| VEGF121 | *VEGFA* |
| Angiogenin | *ANG* |
| C3d | *C3* |
| Coagulation Factor IX | *F9* |
| Coagulation Factor X | *F10* |
| GDF2 | *GDF2* |
| MCP-3 | *CCL7* |
| WNT7A | *WNT7A* |
| Glucagon | *GCG* |
| BID | *BID* |
| C3a | *C3* |
| C3d | *C3* |
| Calcineurin | *PPP3CA PPP3R1* |
| Caspase-2 | *CASP2* |
| Coactosin-like protein | *COTL1* |
| Coagulation Factor V | *F5* |
| Coagulation Factor V | *F5* |
| D-dimer | *FGA FGB FGG* |
| Endoglin | *ENG* |
| Galectin-8 | *LGALS8* |
| GIB | *PLA2G1B* |
| GOT1 | *GOT1* |
| HCC-4 | *CCL16* |
| HCG | *CGA CGB* |
| Hemoglobin | *HBA1 HBB* |
| IgD | *IGHD* |
| Integrin aVb5 | *ITGAV ITGB5* |
| Kininogen, HMW, Two Chain | *KNG1* |
| LIF sR | *LIFR* |
| Lysozyme | *LYZ* |
| MIP-3b | *CCL19* |
| MIS | *AMH* |
| MMP-1 | *MMP1* |
| MMP-13 | *MMP13* |
| Stanniocalcin-1 | *STC1* |
| TF | *F3* |
| EPI | *EREG* |
| AGR2 | *AGR2* |
| annexin I | *ANXA1* |
| annexin II | *ANXA2* |
| ARMEL | *CDNF* |
| ARP19 | *ARPP19* |
| ARTS1 | *ERAP1* |
| ATP synthase beta chain | *ATP5B* |
| C1QBP | *C1QBP* |
| CAPG | *CAPG* |
| Carbonic anhydrase I | *CA1* |
| carbonic anhydrase II | *CA2* |
| CATZ | *CTSZ* |
| cIAP-2 | *BIRC3* |
| CRK | *CRK* |
| DSC3 | *DSC3* |
| Elafin | *PI3* |
| ERP29 | *ERP29* |
| Esterase D | *ESD* |
| FABPE | *FABP5* |
| FAK1 | *PTK2* |
| FCAR | *FCAR* |
| Fibrinogen g-chain dimer | *FGG* |
| GP1BA | *GP1BA* |
| GPC5 | *GPC5* |
| GRN | *GRN* |
| GSTA3 | *GSTA3* |
| hnRNP K | *HNRNPK* |
| HPG- | *HPGD* |
| HRG | *HRG* |
| IF4A3 | *EIF4A3* |
| JAK2 | *JAK2* |
| LG3BP | *LGALS3BP* |
| MMP-14 | *MMP14* |
| MK13 | *MAPK13* |
| MAPK14 | *MAPK14* |
| Mn SOD | *SOD2* |
| Moesin | *MSN* |
| PBEF | *NAMPT* |
| Myokinase, human | *AK1* |
| NCC27 | *CLIC1* |
| NCK1 | *NCK1* |
| PAFAH | *PLA2G7* |
| Peroxiredoxin-5 | *PRDX5* |
| Peroxiredoxin-6 | *PRDX6* |
| phosphoglycerate kinase 1 | *PGK1* |
| PPase | *PPA1* |
| PUR8 | *ADSL* |
| Rb | *RB1* |
| RS3 | *RPS3* |
| S100A7 | *S100A7* |
| sCD163 | *CD163* |
| SEPR | *FAP* |
| SIRT2 | *SIRT2* |
| SPTA2 | *SPTAN1* |
| SSRP1 | *SSRP1* |
| Tropomyosin 1 alpha chain | *TPM1* |
| Trypsin 2 | *PRSS2* |
| TS | *TYMS* |
| TSG-6 | *TNFAIP6* |

**Supplemental Table 2** List of analytes selected from the menu of the Aushon SearchLight multiplex platform

| Ang-2 | MMP-9 |
| --- | --- |
| bFGF | OPN |
| CD40L | PAI-1 |
| E-Selectin | PDGF-AA |
| Fibrinogen | PDGF-AB |
| Fibronectin | PDGF-BB |
| G-CSF | PlGF |
| GM-CSF | SCF |
| HGF | SDF-1b |
| CRP | TGFb1 |
| ICAM-1 | TGFb2 |
| ICAM-3 | TIMP-1 |
| IL-1a | TIMP-2 |
| IL-1b | TNF-a |
| IL-6 | TRAIL |
| IL-8 | TSP-1 |
| IP-10 | VCAM-1 |
| ITAC | VEGF-A |
| KGF | VEGF-C |
| MCP-1 | VEGF-D |
| MIG | VEGFR-1 |
| MMP-1 | VEGFR-2 |
| MMP-2 |  |

**Supplemental Table 3** Baseline patient characteristics by treatment arm in the overall trial population and in the biomarker analysis subgroups

|  | Overall trial population^2^ | | Serum soluble protein biomarker analysis subgroup^1^  (n=73) | Germline SNP biomarker analysis subgroup  (n=202) | Molecular  tumor markers:  IHC-evaluable subgroup^2^  (n=153) | Molecular tumor markers:  VHL-evaluable subgroup  (n=143) |
| --- | --- | --- | --- | --- | --- | --- |
|  | Sunitinib  50 mg/day  Schedule 4/2  (n=146) | Sunitinib  37.5 mg/day  CDD schedule  (n=146) |
| Male | 101 (69) | 89 (61) | 50 (68) | 135 (67) | 97 (63) | 90 (63) |
| Median age,^3^ years (range) | 61 (35–84) | 64 (44–86) | 59 (35–84) | NA | 62 (35–81) | 61 (35–81) |
| Mean weight (SD), kg | 85.7 (21.9) | 85.1 (22.3) | NA | NA | 83.4 (21.4) | 84.0 (21.4) |
| Mean height (SD), cm | 172.9 (9.4) | 170.3 (9.5) | NA | NA | 171.3 (10.2) | 171.3 (10.1) |
| Race, n (%)  White  Black  Asian  Other | 132 (90) 6 (4) 2 (1) 6 (4) | 128 (88) 7 (5) 2 (1) 9 (6) | NA | 178 (88)  10 (5)  3 (1)  11 (5) | 136 (89)  7 (5)  3 (2)  7 (5) | 128 (90)  6 (4)  3 (2)  6 (4) |
| Karnofsky performance status, n (%)  ≥80  <80 | 142 (97)  4 (3) | 129 (88)  17 (12) | 71 (97)  2 (3) | 190 (94)  12 (6) | 142 (93)  11 (7) | 134 (94)  9 (6) |
| Prior nephrectomy,  n (%) | 117 (80) | 113 (77) | 59 (81) | NA | NA | NA |
| Previous radiation therapy,^4^ n (%) | 19 (13) | 15 (10) | NA | NA | NA | NA |
| Risk factors based on published MSKCC data,^5^ n (%)  0 (favorable)  1–2 (intermediate)  ≥ 3 (poor) | 43 (29)  91 (62)  12 (8) | 38 (26)  88 (60)  20 (14) | 24 (33)  42 (58)  7 (10) | 57 (28)  124 (61)  21 (10) | 44 (29)  93 (61)  16 (10) | 41 (29)  88 (62)  14 (10) |
| CDD = continuous daily dosing; SNP = single nucleotide polymorphism ; IHC = immunohistochemistry; VHL = *Von Hippel–Lindau* [gene]*;*  NA = not available; SD, standard deviation; MSKCC = Memorial Sloan-Kettering Cancer Center  ^1^Schedule 4/2 patients only were included in this analysis, and data were missing for one patient.  ^2^Four patients were excluded from the analysis due to inevaluable samples.  ^3^For IHC- and VHL-evaluable subgroups, mean age is reported; in the SNP subgroup, neither median nor mean age were available; however, 120 patients (59%) and 82 patients (41%) were <65 years and ≥65 years, respectively.  ^4^Data not reported for 8 and 11 patients in the Schedule 4/2 and CDD arms, respectively  ^5^Includes low serum hemoglobin level; elevated corrected serum calcium level; elevated serum lactate dehydrogenase level; poor performance status; and interval of <1 year between diagnosis and treatment.^14^ | | | | | | |

**Supplemental Table 4** Soluble protein biomarkers from the SOMAscan platform with differences in baseline levels by extreme of response in patients on Schedule 4/2, selected for unadjusted *p* value comparison ≤0.05

|  |  | Extreme of response (RECIST v1.0) | | | | | |  |
| --- | --- | --- | --- | --- | --- | --- | --- | --- |
| Analyte^a^ | Unit | CR or PR | | *n* | SD <24 weeks or PD | | *n* | *p* value^b^ |
|  |  | Mean | Median |  | Mean | Median |  |  |
| ADAMTS-4 | pg/ml | 247.41 | 238.00 | 27 | 261.13 | 247.00 | 31 | 0.0441 |
| AURKB | pg/ml | 639.04 | 625.00 | 27 | 662.39 | 662.00 | 31 | 0.0242 |
| Afamin | ug/ml | 104.58 | 109.00 | 27 | 79.88 | 79.30 | 31 | 0.0165 |
| Albumin | ug/ml | 1.04 | 1.07 | 27 | 0.90 | 0.94 | 31 | 0.0292 |
| Ang-2 | ng/ml | 29.63 | 29.60 | 27 | 40.93 | 36.70 | 31 | 0.0092 |
| Apo E | ug/ml | 57.27 | 57.10 | 27 | 46.41 | 49.40 | 31 | 0.0079 |
| Apo E3 | ug/ml | 31.82 | 30.90 | 27 | 26.21 | 26.60 | 31 | 0.0058 |
| Apo E4 | ug/ml | 8.23 | 8.15 | 27 | 6.85 | 6.41 | 31 | 0.0303 |
| BGH3 | ug/ml | 2.60 | 2.31 | 27 | 2.10 | 2.19 | 31 | 0.0363 |
| C2 | ug/ml | 20.14 | 20.10 | 27 | 22.51 | 21.60 | 31 | 0.0465 |
| CD36 antigen | ng/ml | 70.49 | 71.30 | 27 | 66.63 | 61.50 | 31 | 0.0499 |
| CD48 | pg/ml | 1,495.93 | 990.00 | 27 | 922.00 | 869.00 | 31 | 0.0419 |
| CTAP-III | ng/ml | 5,280.37 | 5,220.00 | 27 | 6,337.74 | 5,970.00 | 31 | 0.0314 |
| Ck-b-8-1 | pg/ml | 649.15 | 553.00 | 27 | 824.97 | 761.00 | 31 | 0.0111 |
| Coactosin-like protein | ng/ml | 2.55 | 2.52 | 27 | 2.74 | 2.73 | 31 | 0.0357 |
| Contactin-5 | ng/ml | 3.81 | 2.37 | 27 | 2.14 | 2.09 | 31 | 0.0377 |
| Cytochrome c | ng/ml | 2.45 | 2.36 | 27 | 2.73 | 2.55 | 31 | 0.0216 |
| DKK3 | ng/ml | 28.57 | 28.60 | 27 | 26.44 | 25.00 | 31 | 0.0193 |
| Gelsolin | ug/ml | 206.07 | 209.00 | 27 | 181.07 | 181.00 | 31 | 0.0499 |
| IL-1 R AcP | ng/ml | 121.35 | 98.60 | 27 | 101.90 | 88.50 | 31 | 0.0381 |
| IL-11 RA | ng/ml | 3.49 | 3.29 | 27 | 3.58 | 3.15 | 31 | 0.0261 |
| LBP | ug/ml | 22.65 | 18.90 | 27 | 33.65 | 27.10 | 31 | 0.0357 |
| LSAMP | ng/ml | 9.44 | 9.40 | 27 | 8.48 | 7.53 | 31 | 0.0266 |
| MMP-2 | ng/ml | 9.21 | 9.07 | 27 | 8.26 | 7.81 | 31 | 0.0491 |
| MPIF-1 | pg/ml | 521.67 | 445.00 | 27 | 665.58 | 624.00 | 31 | 0.0419 |
| MRC2 | ng/ml | 32.09 | 29.00 | 27 | 27.66 | 26.50 | 31 | 0.0482 |
| NCAM-120 | ng/ml | 230.89 | 220.00 | 27 | 191.24 | 180.00 | 31 | 0.0037 |
| PAI-1 | ng/ml | 29.57 | 23.80 | 27 | 30.20 | 30.60 | 31 | 0.0397 |
| PH | ng/ml | 0.86 | 0.72 | 27 | 0.68 | 0.51 | 31 | 0.0450 |
| RBP | ug/ml | 45.29 | 27.45 | 8 | 15.14 | 9.00 | 12 | 0.0346 |
| SPARCL1 | ng/ml | 369.04 | 367.00 | 27 | 326.16 | 307.00 | 31 | 0.0185 |
| Siglec-9 | pg/ml | 466.59 | 496.00 | 27 | 654.26 | 587.00 | 31 | 0.0384 |
| TARC | pg/ml | 195.03 | 167.00 | 27 | 156.24 | 134.00 | 31 | 0.0383 |
| TIMP-1 | ng/ml | 161.59 | 154.00 | 27 | 196.90 | 176.00 | 31 | 0.0434 |
| TIMP-2 | ng/ml | 124.20 | 124.00 | 27 | 112.51 | 109.00 | 31 | 0.0181 |
| TPSB2 | ng/ml | 1.37 | 1.33 | 27 | 1.07 | 1.01 | 31 | 0.0200 |
| TrkB | ng/ml | 46.51 | 38.50 | 27 | 35.12 | 34.50 | 31 | 0.0303 |
| Trypsin | ng/ml | 12.74 | 9.64 | 27 | 8.81 | 7.60 | 31 | 0.0370 |
| WFKN2 | ng/ml | 12.94 | 13.10 | 27 | 11.90 | 11.80 | 31 | 0.0101 |
| Complement factor H‑related 5 | ug/ml | 2.71 | 2.68 | 27 | 3.32 | 3.13 | 31 | 0.0238 |
| Contactin-1 | ng/ml | 267.37 | 265.00 | 27 | 235.23 | 233.00 | 31 | 0.0491 |
| Kallikrein 5 | pg/ml | 54.96 | 52.90 | 27 | 62.51 | 57.90 | 31 | 0.0171 |
| sFRP-3 | ng/ml | 10.62 | 7.81 | 27 | 6.71 | 6.48 | 31 | 0.0078 |

CR, complete response; FDR, false discovery rate; PD, progressive disease; PR, partial response; RECIST, Response Evaluation Criteria in Solid Tumors; SD, stable disease
^a^ From a menu of ~980 analytes tested (see Supplemental Table 1)
 ^b^ Wilcoxon rank-sum test, with unadjusted *p* value comparison to alpha = 0.05; the adjusted comparison would be to alpha = 0.0000478; at 0.9818, the FDR for these markers was high (i.e. close to 1), indicating low confidence in the observed associations

**Supplemental Table 5** Summary of soluble protein biomarkers from the SOMAscan platform for which the ratios of follow-up (end of treatment) levels to baseline were selected for unadjusted *p* value comparison ≤0.05 and FDR ≤0.05 in patients on Schedule 4/2

| Analyte^a^ | Ratio of  follow‑up: baseline | |  |  |
| --- | --- | --- | --- | --- |
|  | Minimum | Maximum | *p* value^b^ | FDR |
| LG3BP | 0.66 | 2.04 | 0.0006 | 0.0150 |
| Lysozyme | 0.52 | 1.27 | 0.0001 | 0.0051 |
| M-CSF R | 0.52 | 1.22 | 0.0007 | 0.0154 |
| MATN3 | 0.84 | 2.40 | 0.0051 | 0.0480 |
| MBD4 MDC | 0.18 | 2.46 | 0.0001 | 0.0045 |
| MED-1 | 0.74 | 1.07 | 0.0046 | 0.0455 |
| MMP-10 | 0.38 | 1.21 | 0.0036 | 0.0407 |
| Myoglobin | 0.79 | 1.54 | 0.0002 | 0.0078 |
| NG36 | 0.68 | 1.44 | 0.0007 | 0.0154 |
| NKG2D | 0.67 | 1.09 | 0.0041 | 0.0442 |
| OBCAM | 0.60 | 8.37 | 0.0001 | 0.0037 |
| PA2G4 | 0.48 | 4.03 | 0.0007 | 0.0154 |
| PACAP-38 | 0.75 | 1.17 | 0.0052 | 0.0480 |
| PBEF | 0.61 | 1.26 | 0.0032 | 0.0375 |
| PSA | 0.88 | 1.80 | 0.0001 | 0.0042 |
| PTK6 | 0.79 | 1.06 | 0.0003 | 0.0099 |
| Peroxiredoxin-5 | 0.77 | 1.96 | 0.0001 | 0.0051 |
| Properdin | 0.51 | 1.54 | 0.0002 | 0.0055 |
| RPS6KA3 | 0.74 | 1.10 | 0.0006 | 0.0150 |
| SAA | 0.17 | 9.02 | 0.0001 | 0.0042 |
| SPTA2 | 0.61 | 1.47 | 0.0052 | 0.0480 |
| SRCN1 | 0.72 | 1.13 | 0.0012 | 0.0214 |
| Sialoadhesin | 0.83 | 1.55 | 0.0041 | 0.0442 |
| TEC | 0.82 | 1.14 | 0.0032 | 0.0375 |
| TRY3 | 0.84 | 1.36 | 0.0002 | 0.0055 |
| TSP2 | 0.59 | 2.65 | 0.0007 | 0.0154 |
| TXD12 | 0.70 | 3.33 | 0.0052 | 0.0480 |
| Tenascin | 0.75 | 192.74 | 0.0012 | 0.0214 |
| Thyroglobulin | 0.85 | 1.30 | 0.0007 | 0.0154 |
| TrATPase | 0.89 | 1.69 | 0.0014 | 0.0232 |
| Tropomyosin1 alpha chain | 0.55 | 1.77 | 0.0022 | 0.0319 |
| Trypsin | 0.42 | 3.17 | 0.0046 | 0.0455 |
| VEGF | 0.88 | 1.68 | 0.0001 | 0.0045 |
| XTP3A | 0.60 | 2.09 | 0.0016 | 0.0258 |
| ZAP70 | 0.82 | 1.78 | 0.0034 | 0.0397 |
| a2-Macroglobulin | 0.59 | 1.29 | 0.0018 | 0.0288 |
| gpIIbIIIa | 0.68 | 1.05 | 0.0003 | 0.0085 |
| Resistin | 0.49 | 1.97 | 0.0036 | 0.0407 |
| 2B11 | 0.68 | 1.16 | 0.0005 | 0.0144 |
| FCG2B | 0.34 | 1.60 | 0.0044 | 0.0455 |
| GNS | 0.65 | 1.75 | 0.0052 | 0.0480 |
| Lipocalin 2 | 0.31 | 1.86 | 0.0052 | 0.0480 |

| Analyte^a^ | Ratio of follow‑up:baseline | |  |  |
| --- | --- | --- | --- | --- |
|  | Minimum | Maximum | *p* value^b^ | FDR |
| ADAMTS-5 | 0.92 | 1.48 | 0.0029 | 0.0375 |
| AGR2 | 0.62 | 2.26 | 0.0046 | 0.0455 |
| ASAH2 | 0.73 | 3.74 | 0.0041 | 0.0442 |
| ATS13 | 0.46 | 1.57 | 0.0001 | 0.0045 |
| Adiponectin | 0.64 | 2.26 | 0.0010 | 0.0194 |
| Albumin | 0.66 | 1.13 | 0.0014 | 0.0232 |
| C3 | 0.19 | 3.15 | 0.0032 | 0.0375 |
| C5 | 0.90 | 1.83 | 0.0021 | 0.0311 |
| C5b,6 complex | 0.32 | 3.21 | 0.0007 | 0.0154 |
| C9 | 0.46 | 2.46 | 0.0032 | 0.0375 |
| CBG | 0.54 | 1.55 | 0.0024 | 0.0328 |
| CBX5 | 0.88 | 2.11 | 0.0046 | 0.0455 |
| CD5L | 0.43 | 1.55 | 0.0009 | 0.0173 |
| CK-MM | 0.76 | 2.04 | 0.0046 | 0.0455 |
| CLC7A | 0.80 | 1.11 | 0.0010 | 0.0194 |
| CO8A1 | 0.59 | 1.10 | 0.0028 | 0.0364 |
| CRP | 0.13 | 25.32 | 0.0004 | 0.0111 |
| CTACK | 0.94 | 1.63 | 0.0005 | 0.0125 |
| CYTF | 0.36 | 1.11 | 0.0032 | 0.0375 |
| Calpain I | 0.58 | 1.62 | 0.0014 | 0.0232 |
| Calpastatin | 0.43 | 1.37 | 0.0036 | 0.0407 |
| Caspase 3 | 0.22 | 1.21 | 0.0016 | 0.0258 |
| Cathepsin B | 0.68 | 1.97 | 0.0024 | 0.0328 |
| Coagulation Factor V | 0.82 | 1.66 | 0.0003 | 0.0085 |
| Cofilin-1 | 0.78 | 1.66 | 0.0004 | 0.0116 |
| DLRB1 | 0.90 | 1.61 | 0.0024 | 0.0328 |
| ETHE1 | 0.87 | 1.44 | 0.0002 | 0.0065 |
| Ephrin-B3 | 0.66 | 1.09 | 0.0014 | 0.0232 |
| FCGR1 | 0.81 | 1.12 | 0.0024 | 0.0328 |
| Factor B | 0.71 | 1.47 | 0.0032 | 0.0375 |
| FLT3 Ligand | 0.90 | 2.27 | 0.0051 | 0.0480 |
| Galectin-4 | 0.94 | 1.28 | 0.0001 | 0.0053 |
| HPLN1 | 0.90 | 1.52 | 0.0006 | 0.0150 |
| IGFBP-4 | 0.73 | 1.58 | 0.0046 | 0.0455 |
| IL-1 R4 | 0.55 | 4.12 | 0.0028 | 0.0364 |
| IL-17B | 0.86 | 1.57 | 0.0006 | 0.0150 |
| IL-20 | 0.93 | 1.35 | 0.0018 | 0.0288 |
| IL-3 Ra | 0.61 | 1.17 | 0.0008 | 0.0170 |
| IL-6 sRa | 0.61 | 1.28 | 0.0024 | 0.0328 |
| IL-8 | 0.92 | 1.25 | 0.0019 | 0.0288 |
| IL‑22 RA1 | 0.55 | 1.16 | 0.0032 | 0.0375 |
| Kallikrein 6 | 0.70 | 1.53 | 0.0041 | 0.0442 |
| LDH-H1 | 0.57 | 3.14 | 0.0004 | 0.0111 |
| LEAP-1 | 0.25 | 8.27 | 0.0021 | 0.0311 |

FDR, false discovery rate (in which a value close to 1 indicates low confidence in the observed associations)

^a^ From a menu of ~980 analytes tested (see Supplemental Table 1)

^b^ Wilcoxon rank-sum test, with unadjusted *p* value comparison to alpha = 0.05; the adjusted comparison would be to alpha = 0.0000478

**Supplemental Table 6** Soluble protein biomarkers from the SOMAscan platform with differences in ratios of follow-up (end of treatment) levels to baseline by best overall response in patients on Schedule 4/2, selected for unadjusted *p* value comparison ≤0.05

|  | Best overall tumor response (RECIST v1.0) | | | | | |  |
| --- | --- | --- | --- | --- | --- | --- | --- |
| Analyte^a^ | CR or PR | | *n* | SD or PD | | *n* | *p* value^b^ |
|  | Mean ratio | Median ratio |  | Mean ratio | Median ratio |  |  |
| AK1A1 | 0.83 | 0.75 | 8 | 1.15 | 1.07 | 15 | 0.0365 |
| ARI3A | 1.11 | 1.09 | 8 | 0.98 | 0.99 | 15 | 0.0417 |
| Angiotensinogen | 1.43 | 1.28 | 8 | 1.05 | 1.04 | 15 | 0.0319 |
| CATZ | 0.88 | 0.87 | 8 | 1.12 | 1.08 | 15 | 0.0365 |
| CD23 | 0.86 | 0.93 | 8 | 1.05 | 1.02 | 15 | 0.0319 |
| Cathepsin B | 0.97 | 1.02 | 8 | 1.24 | 1.16 | 15 | 0.0076 |
| ECM1 | 1.11 | 1.14 | 8 | 0.88 | 0.86 | 15 | 0.0417 |
| FGF7 | 1.04 | 1.03 | 8 | 0.95 | 0.94 | 15 | 0.0158 |
| FSTL3 | 0.89 | 0.88 | 8 | 1.23 | 1.14 | 15 | 0.0210 |
| Fucosyltransferase 3 | 1.11 | 1.09 | 8 | 0.96 | 0.95 | 15 | 0.0210 |
| HGFA | 1.20 | 1.05 | 7 | 0.96 | 0.87 | 15 | 0.0466 |
| HINT1 | 1.00 | 1.01 | 8 | 0.93 | 0.91 | 15 | 0.0319 |
| I-TAC | 0.66 | 0.67 | 8 | 1.39 | 1.09 | 15 | 0.0066 |
| IL-11 | 1.03 | 0.99 | 8 | 0.94 | 0.93 | 15 | 0.0476 |
| Integrin a1b1 | 0.85 | 0.80 | 8 | 1.34 | 1.23 | 15 | 0.0242 |
| MCM2 | 1.09 | 1.03 | 8 | 0.89 | 0.86 | 15 | 0.0210 |
| MCP-1 | 0.81 | 0.82 | 8 | 1.15 | 1.11 | 15 | 0.0365 |
| MIP-5 | 0.84 | 0.85 | 8 | 1.15 | 1.11 | 15 | 0.0365 |
| OTUB1 | 1.07 | 1.05 | 8 | 0.97 | 0.95 | 15 | 0.0417 |
| PAFAH beta subunit | 0.88 | 0.89 | 8 | 1.05 | 1.01 | 15 | 0.0476 |
| PESC | 1.08 | 1.01 | 8 | 0.91 | 0.92 | 15 | 0.0319 |
| PFD5 | 0.97 | 0.95 | 8 | 1.28 | 1.10 | 15 | 0.0242 |
| PSD7 | 1.30 | 1.14 | 8 | 0.92 | 0.86 | 15 | 0.0476 |
| RAP | 0.94 | 0.92 | 8 | 1.17 | 1.06 | 15 | 0.0319 |
| SARP-2 | 2.15 | 1.05 | 8 | 0.95 | 0.97 | 15 | 0.0417 |
| SDF-1b | 0.76 | 0.79 | 8 | 1.02 | 0.97 | 15 | 0.0365 |
| A2-Macroglobulin | 0.98 | 0.93 | 8 | 0.78 | 0.78 | 15 | 0.0365 |
| sICAM-2 | 0.94 | 0.94 | 8 | 1.02 | 1.01 | 15 | 0.0242 |
| suPAR | 0.76 | 0.76 | 8 | 0.96 | 0.90 | 15 | 0.0365 |

CR, complete response; FDR, false discovery rate; PD, progressive disease; PR, partial response; RECIST, Response Evaluation Criteria in Solid Tumors; SD, stable disease
^a^ From a menu of ~980 analytes tested (see Supplemental Table 1)
 ^b^ Wilcoxon rank-sum test, with unadjusted *p* value comparison is to alpha = 0.05; the adjusted comparison would be to alpha = 0.0000478; at 0.9657, the FDR for these markers was high (i.e. close to 1), indicating low confidence in the observed associations

**Supplemental Table 7 Soluble protein biomarkers from the SOMAscan platform with differences in ratios of follow-up (end of treatment) levels to baseline by extreme of response in patients on Schedule 4/2, selected for unadjusted *p* value comparison of ≤0.05**

|  | Extreme of response (RECIST v1.0) | | | | | |  |
| --- | --- | --- | --- | --- | --- | --- | --- |
| Analyte^a^ | CR or PR | | *n* | SD <24 weeks or PD | | *n* | *p* value^b^ |
|  | Mean ratio | Median ratio |  | Mean ratio | Median ratio |  |  |
| AK1A1 | 0.83 | 0.75 | 8 | 1.28 | 1.15 | 9 | 0.0315 |
| ARI3A | 1.11 | 1.09 | 8 | 0.95 | 0.94 | 9 | 0.0315 |
| Aflatoxin B1 aldehyde reductase | 0.98 | 0.95 | 8 | 1.62 | 1.54 | 9 | 0.0119 |
| Angiotensinogen | 1.43 | 1.28 | 8 | 0.94 | 1.02 | 9 | 0.0176 |
| CD23 | 0.86 | 0.93 | 8 | 1.11 | 1.02 | 9 | 0.0458 |
| CRK | 0.95 | 0.99 | 8 | 1.11 | 1.11 | 9 | 0.0260 |
| Catalase | 0.95 | 0.82 | 8 | 1.24 | 1.19 | 9 | 0.0458 |
| Cathepsin B | 0.97 | 1.02 | 8 | 1.26 | 1.19 | 9 | 0.0119 |
| DRR1 | 1.07 | 1.04 | 8 | 0.98 | 1.00 | 9 | 0.0260 |
| FGF7 | 1.04 | 1.03 | 8 | 0.96 | 0.95 | 9 | 0.0380 |
| FSTL3 | 0.89 | 0.88 | 8 | 1.28 | 1.10 | 9 | 0.0315 |
| Fucosyltransferase 3 | 1.11 | 1.09 | 8 | 0.90 | 0.90 | 9 | 0.0079 |
| HGFA | 1.20 | 1.05 | 7 | 0.89 | 0.83 | 9 | 0.0148 |
| HINT1 | 1.00 | 1.01 | 8 | 0.92 | 0.90 | 9 | 0.0458 |
| HPLN1 | 1.24 | 1.27 | 8 | 1.01 | 0.98 | 9 | 0.0260 |
| I-TAC | 0.66 | 0.67 | 8 | 1.57 | 1.26 | 9 | 0.0145 |
| Integrin a1b1 | 0.85 | 0.80 | 8 | 1.51 | 1.37 | 9 | 0.0380 |
| MCM2 | 1.09 | 1.03 | 8 | 0.90 | 0.86 | 9 | 0.0260 |
| MIP-5 | 0.84 | 0.85 | 8 | 1.17 | 1.12 | 9 | 0.0458 |
| Mn SOD | 1.07 | 1.08 | 8 | 0.82 | 0.84 | 9 | 0.0315 |
| PAFAH beta subunit | 0.88 | 0.89 | 8 | 1.12 | 1.10 | 9 | 0.0214 |
| PESC | 1.08 | 1.01 | 8 | 0.93 | 0.92 | 9 | 0.0458 |
| PFD5 | 0.97 | 0.95 | 8 | 1.40 | 1.12 | 9 | 0.0260 |
| PH | 1.07 | 0.99 | 8 | 1.28 | 1.19 | 9 | 0.0458 |
| PSD7 | 1.30 | 1.14 | 8 | 0.90 | 0.86 | 9 | 0.0458 |
| Peroxiredoxin 2 | 1.13 | 1.10 | 8 | 0.98 | 0.95 | 9 | 0.0214 |
| Peroxiredoxin-6 | 0.98 | 0.84 | 8 | 1.44 | 1.09 | 9 | 0.0458 |
| SHP-2 | 1.02 | 0.95 | 8 | 1.28 | 1.20 | 9 | 0.0380 |
| STX1a | 1.02 | 1.02 | 8 | 0.96 | 0.96 | 9 | 0.0458 |
| TNF sR-I | 0.97 | 0.94 | 8 | 1.18 | 1.10 | 9 | 0.0380 |
| TNF sR-II | 0.85 | 0.86 | 8 | 1.37 | 1.25 | 9 | 0.0315 |
| TNR4 | 1.06 | 1.02 | 8 | 0.95 | 0.90 | 9 | 0.0458 |
| Trypsin | 0.69 | 0.67 | 8 | 1.21 | 0.99 | 9 | 0.0145 |
| XPNPEP1 | 0.94 | 0.95 | 8 | 1.08 | 1.06 | 9 | 0.0458 |
| sICAM-2 | 0.94 | 0.94 | 8 | 1.02 | 1.01 | 9 | 0.0457 |
| suPAR | 0.76 | 0.76 | 8 | 1.03 | 1.04 | 9 | 0.0260 |

CR, complete response; FDR, false discovery rate; PD, progressive disease; PR, partial response; RECIST, Response Evaluation Criteria in Solid Tumors; SD, stable disease
^a^ From a menu of ~980 analytes tested (see Supplemental Table 1)
 ^b^ Wilcoxon rank-sum test, with unadjusted *p* value comparison to alpha = 0.05; the adjusted comparison would be to alpha = 0.0000478; at 1.000, the FDR for these markers was high, indicating low confidence in the observed associations
